# Lactate Dehydrogenase C4 Accelerates Triple‐Negative Breast Cancer Progression by Promoting Acetyl‐CoA Acyltransferase 2 Lactylation to Increase Free Fatty Acid Accumulation

**DOI:** 10.1002/advs.202511849

**Published:** 2025-08-14

**Authors:** Zhaolei Cui, Chaoqiang Zheng, Yingying Lin, Zhenzhou Xiao, Yanhong Li, Wei Peng, Shijie He, Anyang Li, Xiufeng Wu, Yan Chen, Yang Sun

**Affiliations:** ^1^ Laboratory of Biochemistry and Molecular Biology Research Department of Laboratory Medicine Clinical Oncology School of Fujian Medical University Fujian Cancer Hospital Fuzhou 350014 China; ^2^ Department of Gynecology Clinical Oncology School of Fujian Medical University Fujian Cancer Hospital Fuzhou 350014 China; ^3^ Department of Breast Surgical Oncology Clinical Oncology School of Fujian Medical University Fujian Cancer Hospital Fuzhou 350014 China

**Keywords:** acetyl‐CoA acyltransferase 2, free fatty acids, lactylation, lactate dehydrogenase C4, triple‐negative breast cancer

## Abstract

Lactate‐induced protein lysine (K) lactylation is inherently connected to cellular metabolism and is implicated in oncogenesis. As a crucial glycolytic enzyme in lactate metabolism, lactate dehydrogenase C4 (LDHC4) has undefined yet potentially significant biological functions and mechanistic roles in triple‐negative breast cancer (TNBC) that warrant further investigation. This study aims to determine whether and how LDHC4 affects TNBC progression by regulating protein lactylation. LDHC4 expression in human TNBC tissues and adjacent nontumor tissues is analyzed through immunoblotting and immunohistochemistry (IHC). Functional experiments verified the biological features of LDHC4 in human TNBC cells both in vitro and in vivo (subcutaneous, orthotopic, and pulmonary metastatic mouse models). 4D label‐free lactylproteome expression analysis (4D‐LFQP‐LA), immunoblotting, and immunoprecipitation are utilized to confirm lactylation at specific lysine sites in acetyl‐CoA acyltransferase 2 (ACAA2) following LDHC4 induction. Targeted lipidomic analysis is performed to characterize ACAA2‐induced metabolite alterations. Immunoblotting, immunofluorescence, and transmission electron microscopy are performed to investigate the mechanisms underlying LDHC4‐induced ACAA2 lactylation and tumor progression in TNBC. LDHC4 expression is upregulated in TNBC, and LDHC4 is an independent predictive factor for prognosis. Both in vitro and in vivo experiments demonstrated that LDHC4 promotes TNBC progression. Subsequent mechanistic investigation revealed that LDHC4 enhances the lactylation of ACAA2 at K214, resulting in increased ACAA2 catalytic activity. This increase in ACAA2 activity accelerates fatty acid (FA) metabolism, promotes TNBC progression both in vitro and in vivo, and leads to increased free fatty acid (FFA) generation and accumulation. The increase in FFAs in turn induces autophagy and promotes cell cycle activity in TNBC cells, thereby promoting TNBC progression. The findings reveal a novel pathway through which LDHC4 induces ACAA2 lactylation to regulate FA metabolism in TNBC cells, thus promoting TNBC progression, highlighting the critical role of LDHC4 in TNBC progression.

## Graphical abstract

Diagram of the reprogramming of FA metabolism mediated by LDHC4‐induced ACAA2 activity to promote TNBC progression. LDHC4 drives TNBC progression by enhancing ACAA2‐mediated fatty acid production. The resulting accumulation of FFAs stimulates autophagy and accelerates cell cycle progression, thereby promoting tumor growth.

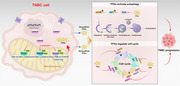

## 1 Introduction

Triple‐negative breast cancer (TNBC), a subtype of breast cancer (BC) characterized by the absence of oestrogen receptor (ER), progesterone receptor (PR), and human epidermal growth factor receptor 2 (HER2) expression, accounts for ≈15–20% of all BC cases.^[^ ^1^ ^]^ TNBC presents substantial clinical challenges not observed with other BC subtypes, including an increased propensity for recurrence and metastasis, a shorter median survival time, and a generally poorer prognosis.^[^ ^2^ ^]^ Therefore, it is imperative to achieve a clearer understanding of TNBC growth and metastasis mechanisms in order to find novel biomarkers to predict TNBC prognosis and potential therapeutic targets.

Cancer‐testis antigens (CTAs) hold great promise as potential treatment targets and biomarkers in BC.^[^ ^3^ ^]^ Lactate dehydrogenase isozyme C4 (LDHC4), which is encoded by the lactate dehydrogenase C (*LDHC*) gene, is expressed exclusively in the testis and some types of tumors; therefore, LDHC4 is classified as a CTA.^[^ ^4^ ^]^ The restricted expression pattern of LDHC4 also makes this protein attractive as a potential target for immunotherapy in cancer, especially BC.^[^ ^5^ ^]^ Previous research indicates that LDHC4 is important for the development and progression of malignant tumors such as lung adenocarcinoma (LUAD),^[^ ^6^ ^]^ and renal cell carcinoma (RCC).^[^ ^7^ ^]^ Our group previously reported that LDHC4 expression is upregulated and also inversely associated with the overall survival (OS) rates in patients with hepatocellular carcinoma (HCC),^[^ ^8^ ^]^ LUAD,^[^ ^9^ ^]^ or non‐TNBC.^[^ ^10^ ^]^ However, the specific role as well as the underlying mechanisms of LDHC4 in the development of TNBC remains unclear.

Metabolic reprogramming, including alterations in glucose metabolism, fatty acid (FA) metabolism, and amino acid metabolism, is a hallmark of cancer cells that distinguishes them from noncancerous cells.^[^ ^11^ ^]^ Indeed, TNBC cells undergo metabolic reprogramming characterized by increased glycolysis, tricarboxylic acid cycle activity, glutaminolysis, and FA biosynthesis.^[^ ^12^ ^]^ These metabolic alterations in TNBC cells reshape the tumor microenvironment to promote angiogenesis and inhibit host immune defenses, thereby accelerating tumor progression.^[^ ^13^ ^]^ In addition to changes in the tumor microenvironment due to metabolic alterations, tumor cells can facilitate signal transduction during metabolic processes through the chemical modification of both histones and nonhistone proteins.^[^ ^14^ ^]^ The posttranslational modification of lysine (K) residues on histones or nonhistone proteins plays pivotal roles in cellular metabolism and regulation of the tumor microenvironment.^[^ ^15^ ^]^ Among the modifications that occur at K residues is protein lactylation, a posttranslational modification that is positively regulated by lactate, a metabolic product of glycolysis.^[^ ^16^ ^]^ Protein lactylation has been demonstrated to be inherently connected to cellular metabolism and is implicated in oncogenesis of various cancers, such as ocular melanoma,^[^ ^17^ ^]^ prostate cancer,^[^ ^18^ ^]^ colorectal cancer,^[^ ^19^ ^]^ and gastric cancer,^[^ ^20^ ^]^ among others. Intrigulingly, it has been reported that lactate dehydrogenase A (LDHA) is a key enzyme that catalyzes protein lactylation.^[^ ^21^ ^]^ For example, research by Hou and colleagues indicated that KCNK1 (potassium two pore domain channel subfamily K member 1) increased the glycolysis and lactate production in BC cells by binding to and activating LDHA and that LDHA ultimately promoted histone lysine lactylation to induce the expression of a series of downstream genes.^[^ ^21a^ ^]^ As a LDH family member, LDHC4 also plays a crucial role in glycolysis, catalyzing the interconversion of lactate and pyruvate.^[^ ^22^ ^]^ However, whether LDHC4 regulates protein lactylation in TNBC to induce metabolic reprogramming warrants further investigations.

Here, based upon 4D label‐free lactylproteome expression analysis (4D‐LFQP‐LA)^[^ ^23^ ^]^ and cell function experiments, we show for the first time that LDHC4 promotes the lactylation of acetyl‐CoA acyltransferase 2 (ACAA2), which is involved in FA β‐oxidation, at the K214 residue and thereby increases the catalytic activity of ACAA2. This change in ACAA2 catalytic activity leads to the accumulation of intermediate FFA metabolites and ultimately promotes TNBC progression. Our findings identify a novel molecular mechanism of LDHC4‐induced protein lactylation in TNBC, providing new experimental evidence for further in‐depth research into lactylation treatment strategies and metabolically targeted therapies in TNBC.

## 2 Results

### 2.1 LDHC4 is Highly Expressed in TNBC and Negatively Correlates with Patient Survival

**Figure 1:**
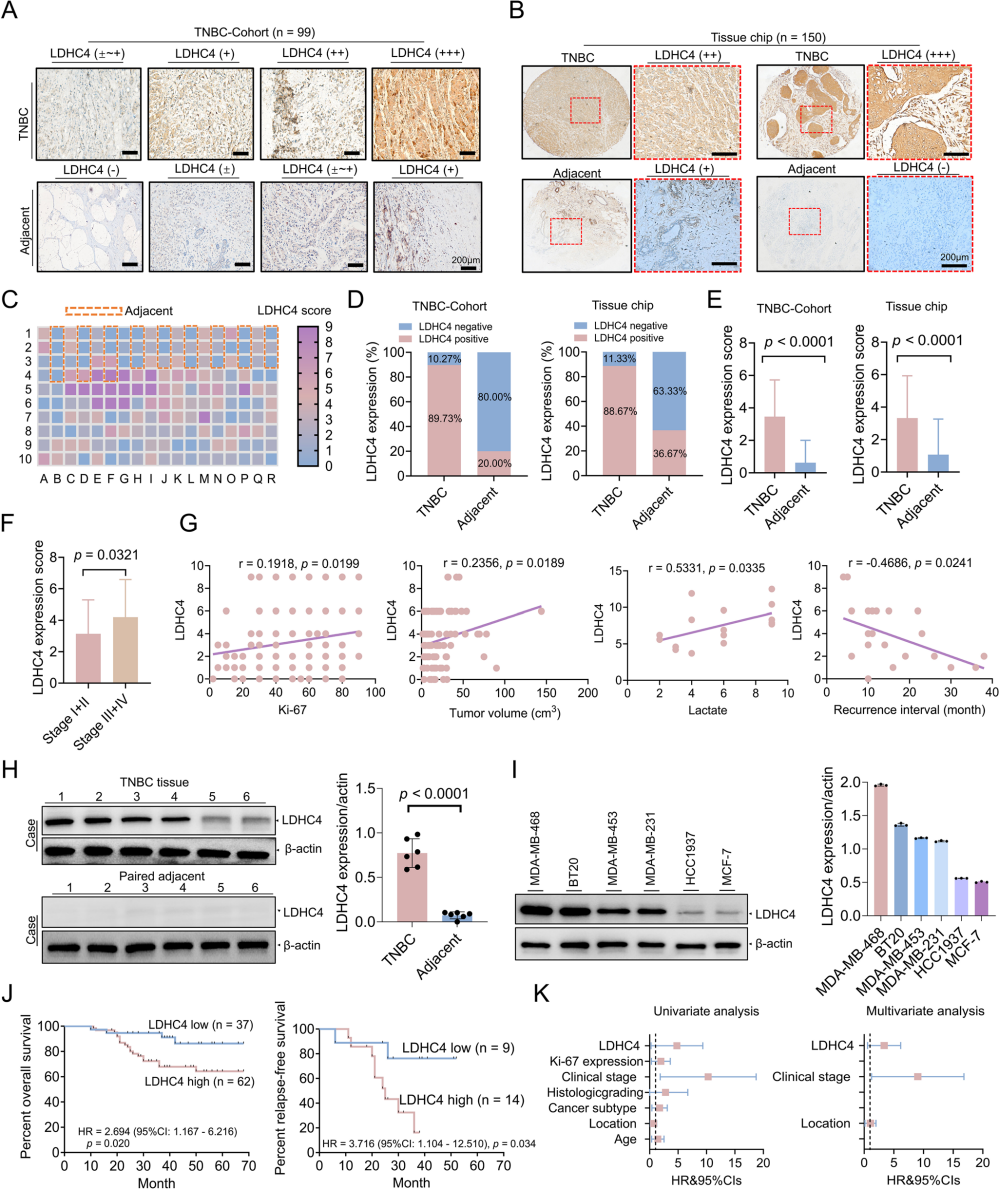
Elevated LDHC4 expression in TNBC is associated with unfavourable prognosis. A,B) Expression status of LDHC4 expression in TNBC tissue cells analyzed based on the individual TNBC‐Cohort (TNBC, n = 99; adjacent, n = 99) and commercial tissue chip (TNBC, n = 150; adjacent, n = 30) by immunohistochemistry (IHC). C) Tissue microarrays show the LDHC4 expression status in TNBC and adjacent nontumor tissues (TNBC, n = 150; adjacent, n = 30); A higher expression score represents higher LDHC4 expression. D,E) LDHC4 expression levels and positivity rates in TNBC tissues and adjacent noncancer tissues. F) Analysis of LDHC4 levels in relation to clinical stage. G) Analysis of the relationship between LDHC4 levels and various clinicopathological factors. Correlation studies revealed positive associations between LDHC4 expression and both Ki‐67 and plasma lactate concentrations. H) Detailed quantitative evaluation of LDHC4 in TNBC instances (n = 6), assessed via immunoblotting and normalized to β‐actin levels. I) Expression of LDHC4 in BC cell lines. J) Kaplan‐Meier analysis of overall survival (OS) and relapse‐free survival (RFS) in TNBC patients with low and high LDHC4 levels. K) A forest plot demonstrating the independent impact of LDHC4 levels on OS among TNBC patients. All data are presented as mean ± SD. Statistical analysis was performed with Student's *t*‐test or the Mann – Whitney test in E, F, and H; Pearson's correlation coefficient or Spearman correlation coefficient analysis in G; log‐rank test in J; and univariate and multivariate regression analyses in K. LDHC4, lactate dehydrogenase C4; TNBC, triple negative breast cancer.

To determine whether LDHC4 is involved in TNBC progression, we assessed the expression and the prognostic value of LDHC4 in TNBC through high‐throughput tissue microarray analysis (TNBC, n = 150; paired adjacent, n = 30) and a TNBC cohort study (TNBC, n = 99; paired adjacent, n = 99), respectively (**Figure** **1**A–C). The positive LDHC4 expression rate was 89.73% in TNBC tissues but only 20% in adjacent nontumor tissues; the average LDHC4 expression level in tumor tissues, as assessed by immunohistochemistry (IHC) scoring, was significantly greater than that in adjacent nontumor tissues (Figure 1D,E); similarly, high‐throughput IHC analysis of tissue microarrays further confirmed that both the LDHC4 positivity rate and average LDHC4 expression level were elevated in tumor tissues compared with adjacent nontumor tissues (Figure 1D,E). Subgroup analyses to determine whether LDHC4 expression correlates with disease severity revealed that LDHC4 expression was lower in patients with stage I‐II disease than in those with stage III–IV disease (Figure 1F). LDHC4 expression was positively correlated with Ki‐67 positivity (a tumor proliferation index with a cut‐off value of 14% in BC^[^ ^24^ ^]^), tumor volume, and lactate levels and negatively correlated with recurrence interval (Figure 1G). Additionally, Chi‐square analysis revealed a significant association between Ki‐67 index and LDHC4 expression (Supplementary Tables S1–S2, Supporting Information). The above results indicated that LDHC4 is involved in TNBC progression and associated with lactate production. Consistently, immunoblotting confirmed the increased expression of LDHC4 in TNBC tissues and BC cell lines (Figure 1H,I). Then, we evaluated the efficacy of LDHC4 in forecasting survival in TNBC patients. Kaplan‐Meier analysis revealed that TNBC patients with low LDHC4 expression had better OS (hazard ratio [HR] = 2.694, 95% confidence interval [CI]: 1.167‐6.216, *p* = 0.020) and relapse‐free survival (RFS) (HR = 3.716, 95%CI: 1.104 – 12.510, *p* = 0.034) than did those with high LDHC4 expression (Figure 1J). Cox univariate and multivariate regression analyses identified LDHC4 as an independent prognostic factor for TNBC (Figure 1K). Collectively, these findings suggest that LDHC4 may be considered a potential biomarker for predicting survival in patients with TNBC.

### 2.2 LDHC4 Promotes TNBC Progression in Vitro and in Vivo

**Figure 2:**
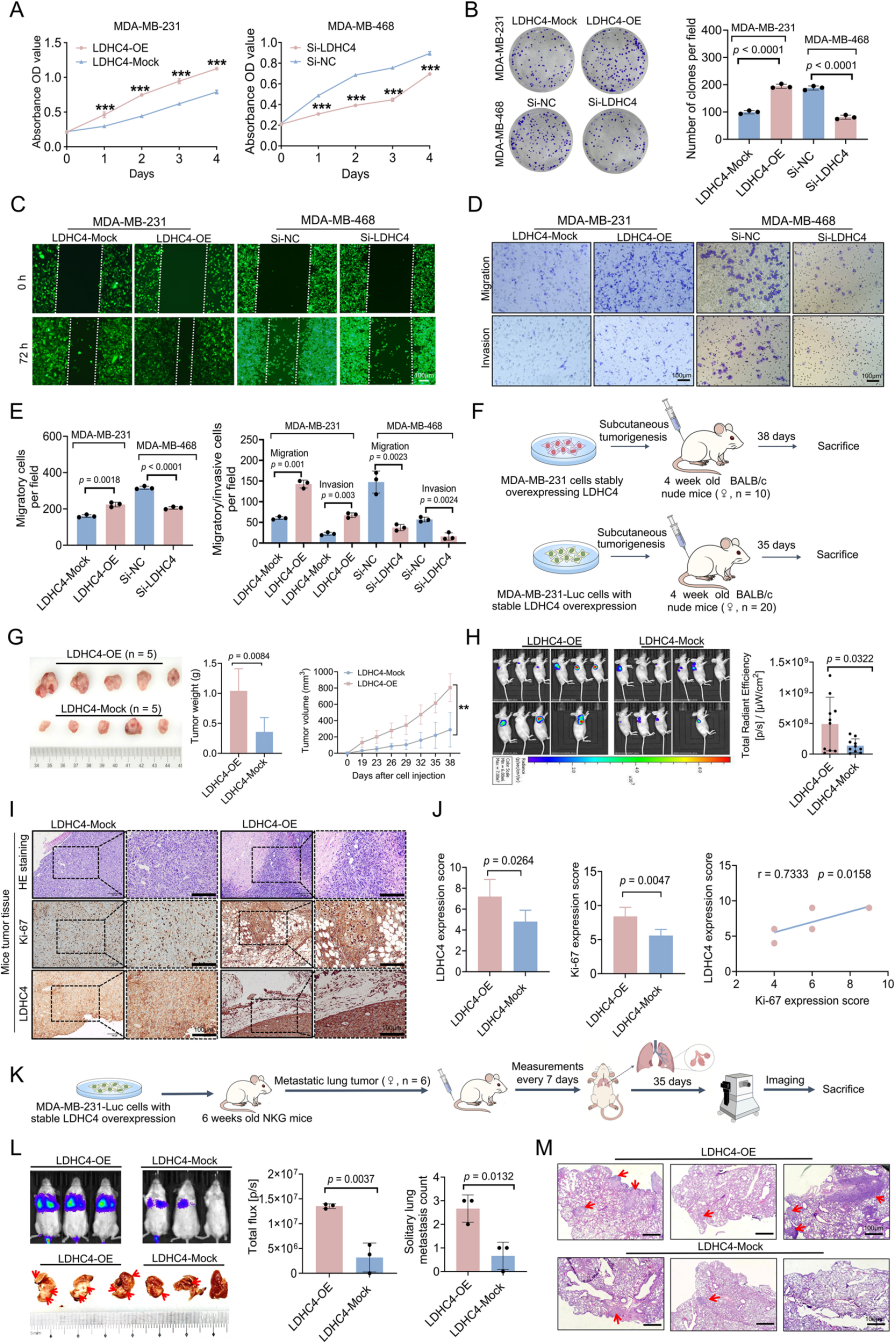
LDHC4 promotes the invasion, migration, and proliferation of TNBC cells in vitro and in vivo. A) CCK‐8 assays were utilized to determine cell viability. B) Colony formation ability. C–E) Migration and invasion capacity were evaluated via wound healing and transwell assays, respectively, of MDA‐MB‐231 and MDA‐MB‐468 cell lines stably infected with lentivirus mediated LDHC4‐OE, ‐Mock, Si‐LDHC4, or Si‐NC vector, and all images were photographed at 0 or 72 h. F,G) Images of xenograft tumors derived from MDA‐MB‐231 cells. Each experimental group consisted of five mice, and the tumor weight and volume were measured and used to generate growth curves. H) In vivo imaging of subcutaneous tumor‐bearing mice derived from MDA‐MB‐231 cells infected with the LDHC4‐OE or ‐Mock vector carrying the luciferase gene. n = 10 mice per group. The evaluation was performed using the Perkin Elmer (Lumina LT) system, and the total radiant efficiency was measured as [p/s]/[µW cm^−2^]. I,J) Further analysis of LDHC4 and Ki‐67 expression in tumor tissues by IHC and the correlation between their expression; HE staining was used to evaluate tumor formation. K–M) Lung metastasis imaging and tumor burden assessment in female NKG mice (n = 3 per group) with histopathological confirmation by HE staining. The red arrow indicates the tumor. All the results are expressed as the mean ± SD. Student's *t*‐test or the Mann – Whitney test was performed in A, B, E, G, H, J, and L, and Spearman correlation coefficient analysis was used to test the correlation of LDHC4 and Ki‐67 in J. **p < 0.01, ***p < 0.001.

To determine whether LDHC4 can influence TNBC cell phenotypes, we overexpressed LDHC4 in MDA‐MB‐231 cells, which have relatively low endogenous LDHC4 expression, and knocked down LDHC4 in MDA‐MB‐468 cells, which exhibit relatively high endogenous LDHC4 expression (Figure 1I). We first evaluated the growth and proliferation of MDA‐MB‐231 and MDA‐MB‐468 cells using CCK‐8 and colony formation assays. In MDA‐MB‐231 cells, upregulation of LDHC4 induced increased proliferation and colony size relative to mock control treatment, whereas LDHC4 knockdown diminished colony number (**Figure** **2**A,B). We subsequently assessed the migration and invasion capabilities of MDA‐MB‐231/LDHC4‐OE and MDA‐MB‐468/Si‐LDHC4 cells. Cells overexpressing LDHC4 exhibited marked promotion of cell migration and invasion compared with the LDHC4‐Mock cells, whereas the depletion of LDHC4 markedly reduced cell migration and invasion compared with Si‐NC treatment, as demonstrated in scratch and transwell assays (Figure 2C–E). To corroborate these findings in vivo, we utilized a xenograft model of TNBC (Figure 2F). Nude mice were injected with LDHC4‐overexpressing (LDHC4‐OE) MDA‐MB‐231 cells, LDHC4‐knockdown (Si‐LDHC4) MDA‐MB‐468 cells, or cells harboring the corresponding mock vectors. The tumors from the LDHC4‐OE group were greater in volume and weight than those from the LDHC4‐Mock group (Figure 2G). In contrast, Si‐LDHC4 MDA‐MB‐468 cells failed to form tumors. In vivo imaging of subcutaneous tumor models revealed that the luciferase fluorescence signal was stronger in the LDHC4‐OE group than in the LDHC4‐Mock group (Figure 2H). Furthermore, IHC analysis of the xenograft tumors revealed that the expression of both LDHC4 and Ki‐67 was greater in LDHC4‐OE tumors than in LDHC4‐Mock tumors (Figure 2I,J), indicating a positive correlation between the expression of LDHC4 and Ki‐67 (Figure 2J). In vivo pulmonary metastasis assays demonstrated that LDHC4 significantly enhances the metastatic potential of MDA‐MB‐231 cells to the lungs (Figure 2K–M). Collectively, these results suggest that LDHC4 accelerates TNBC progression both in vitro and in vivo.

### 2.3 LDHC4 Promotes the Lactylation of the FA Metabolism Enzymes

**Figure 3:**
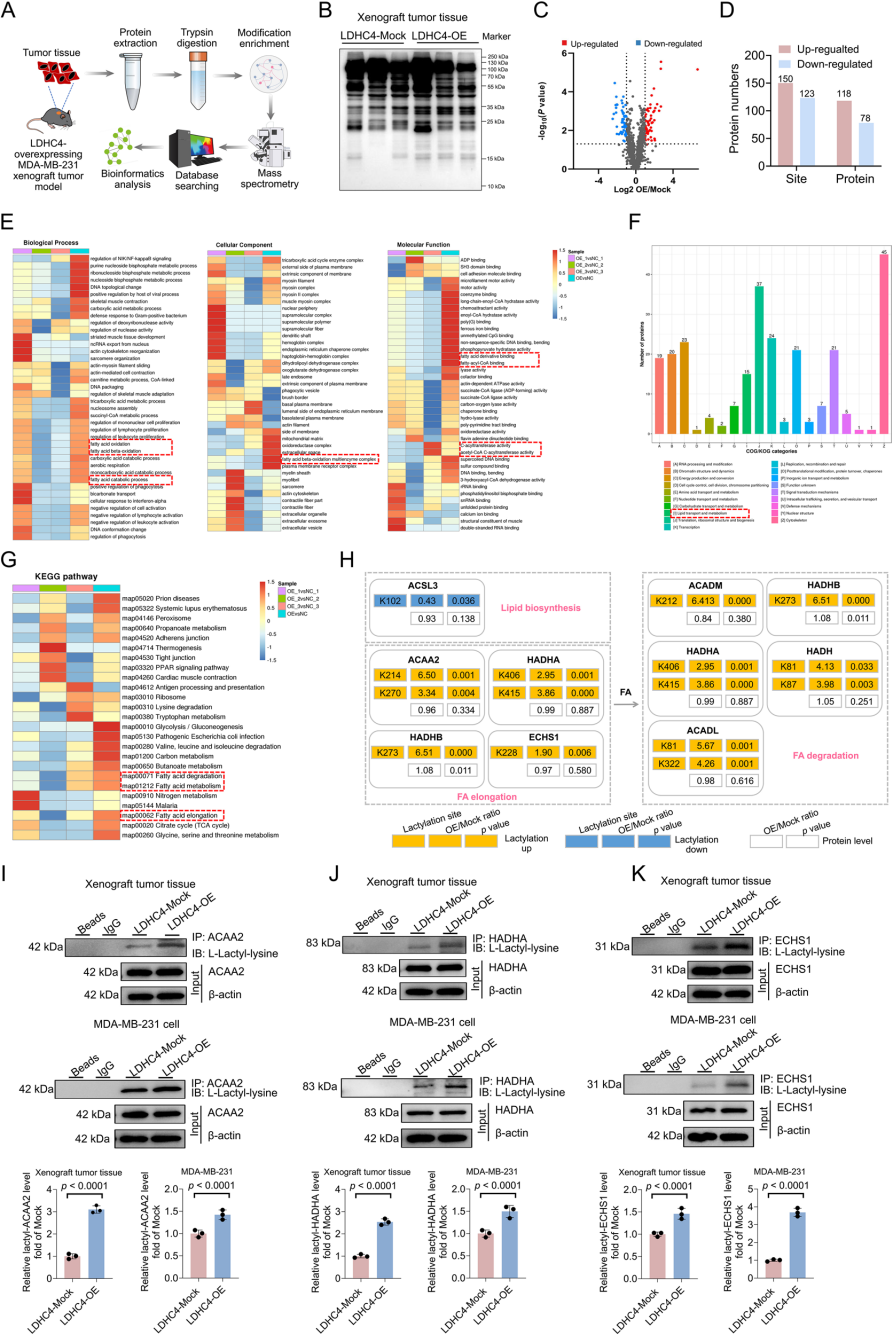
Differential protein lactylation in LDHC4‐OE animal tumors was determined using 4D‐LFQP‐LA. A) Illustration of the 4D‐FLQP‐LA analytical workflow. B) Detection of lactylated proteins in LDHC4‐OE and ‐Mock mouse tumor tissues using a pan‐lactylation antibody (Anti‐L‐Lactyl Lysine Rabbit mAb; PTMBIO, China). C) A volcano plot illustrates differentially lactylated proteins in TNBC. D) Sorting of lactylated proteins in TNBC, arranged according to effect size and statistical significance. E) Gene Ontology (GO) analysis was applied to functionally categorize the lactylated proteins. The red outline indicates enriched components related to FA metabolism. F) Classification was carried out based on Clusters of Orthologous Groups/Eukaryotic Orthologous Groups (COG/KOG) analysis. The red box indicates the categories associated with lipid transport and metabolism. G) Kyoto Encyclopedia of Genes and Genomes (KEGG) pathway analysis showed that differentially lactylated proteins may be involved in processes such as FA elongation/degradation, and FA metabolism, which are indicated with red dashed boxes. H) Overview of altered proteins implicated in FA metabolism pathways based on integrated lactylome analysis. Changes in the protein and lactylation levels of LDHC4‐OE in comparison with ‐Mock are indicated. I–K) Coimmunoprecipitation (co‐IP) and immunoblotting confirmed the increased lactylation of ACAA2, HADHA, and ECHS1 in LDHC4‐OE animal tumors and LDHC4‐OE MDA‐MB‐231 cell lines. All data are presented as mean ± SD. Student's *t*‐test or the Mann – Whitney test was performed in I, J, and K. 4D‐LFQP‐LA: 4D label‐free lactylproteome expression analysis.

Given that LDHC4 is a lactate‐related glycolytic enzyme, we next aimed to determine whether LDHC4 promotes TNBC progression via lactylation. To this end, we first evaluated global lactylation. Tumor tissues generated from LDHC4‐OE and ‐Mock MDA‐MB‐231 cells in three pairs of nude mice each were analysed via 4D‐LFQP‐LA (**Figure** **3**A). Immunoblotting with a pan‐lactylation antibody revealed the expression profiling of lactylated proteins (Figure 3B). After adjustment for fold change and *p* value, significant differences in the lactylation of 196 proteins and 273 lactylation sites were observed; specifically, LDHC4 overexpression was associated with increased lactylation at 150 sites on 118 proteins and decreased lactylation at 123 sites on 78 proteins (Figure 3C,D).

GO enrichment analysis was performed to further clarify the molecular functions, cellular components, and biological processes enriched in the differentially lactylated proteins. The results identified 111 proteins involved in FA metabolic processes (fatty acid oxidation, fatty acid beta‐oxidation, fatty acid catabolic process, fatty acid beta‐oxidation multienzyme complex, fatty‐acyl‐CoA binding, fatty acid derivative binding, etc.) and 66 proteins with catalytic activity (acetyl‐CoA C‐acyltransferase activity, C‐acyltransferase activity, etc.) (Figure 3E). COG/KOG categorization revealed a total of 23 proteins involved in energy production and conversion and 15 proteins involved in lipid transport and metabolism that were lactylated upon LDHC4 overexpression (Figure 3F). KEGG enrichment analysis revealed that the differentially lactylated proteins were predominantly enriched in FA elongation (4 proteins), FA degradation (7 proteins), and FA metabolic signaling (7 proteins) (Figure 3G).

On the basis of the above enrichment analyses, we focused our subsequent research on the potential role of LDHC4 in regulating FA metabolism in TNBC through protein lactylation. Integration of the lactylproteome and proteome analyses revealed increased lactylation (total protein level not altered) at five lysine (K) residues in three enzymes involved in FA elongation, ACAA2 (K214, K270), HADHA (hydroxyacyl‐CoA dehydrogenase trifunctional multienzyme complex subunit alpha) (K406, K415), and ECHS1 (enoyl‐CoA hydratase, short chain 1) (K228) (Figure 3H). Further validation through coimmunoprecipitation (co‐IP) followed by immunoblotting with a pan‐lactylation antibody confirmed an increase in ACAA2, HADHA, and ECHS1 lactylation in LDHC4‐OE xenograft tumors relative to the ‐Mock tumors (Figure 3I–K), consistent with the results of the lactylproteome expression analysis of the xenograft tumors. Similar results were also obtained for the LDHC4‐OE/Mock MDA‐MB‐231 cells (Figure 3I–K), indicating consistency between the in vitro and in vivo results with respect to the increased lactylation of ACAA2, HADHA, and ECHS1 in TNBC. All of these results supported the findings from our in‐depth investigation of the impacts of LDHC4‐induced ACAA2, HADHA, and ECHS1 lactylation on FA metabolic reprogramming in TNBC.

### 2.4 K214 Lactylation Affects the Catalytic Activity of the Enzyme ACAA2

**Figure 4:**
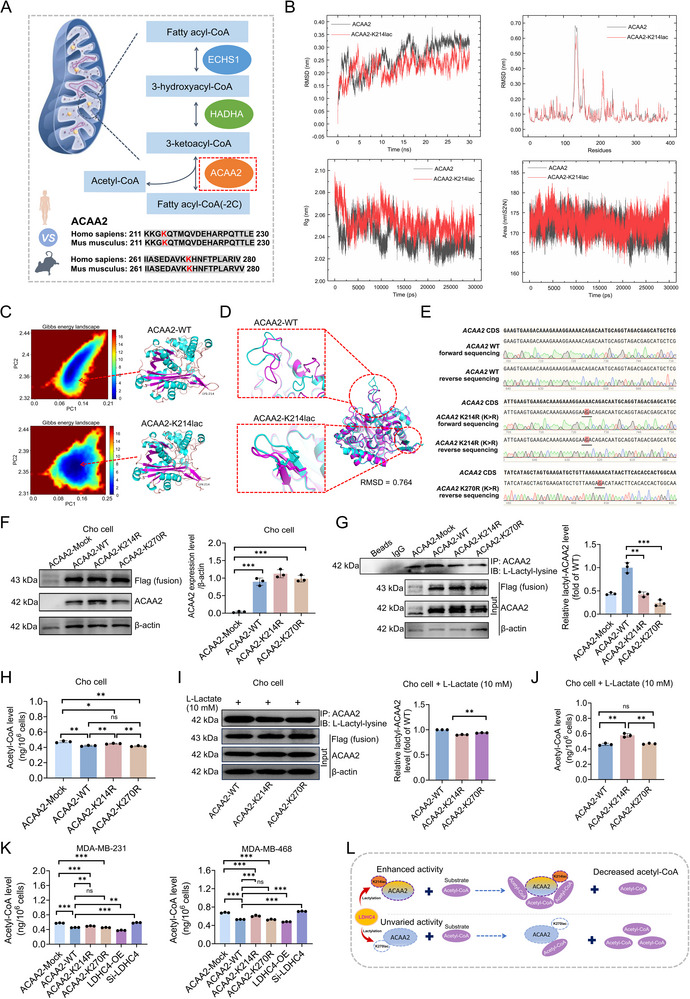
Impact of ACAA2 lactylation at K214 and K270 on ACAA2 catalytic activity. A) ACAA2 plays a central role in the catalytic chain of three FA metabolism regulatory enzymes and participates in the final catalytic step of FA β‐oxidation. The lysine sites in three FA metabolism regulatory enzymes that undergo lactylation are highly conserved between humans and mice. B) Comparison of molecular dynamics (MD) simulation parameters for wild‐type ACAA2 versus ACAA2‐K214lac. C) MD simulations (30 ns) were conducted to compare wild‐type ACAA2 and ACAA2‐K214lac. D) The energy‐minimized structures of wild‐type ACAA2 and ACAA2‐K214lac were subjected to structural alignment. E) Construction and identification of mutant and wild‐type plasmids to express ACAA2 with residues K214 and K270 mutated to arginine residues in the mutant plasmids. F,G) Coimmunoprecipitation (co‐IP) and immunoblotting were used to assess the impact of transfection with the various plasmids on ACAA2 protein levels and lactylation in engineered Cho cells. H) The expression of ACAA2‐K214R results in higher levels of the substrate acetyl‐CoA than that upon expression of ACAA2‐WT or the ‐K270R variant. I) Lactate induces the lactylation of ACAA2. Cho cells were treated with sodium L‐lactate (10 mm) treatment for 24 h. J) Unlike ACAA2‐K270R and ‐WT, the ‐K214R variant showed no lactate‐induced activity enhancement. K) Transfection of the ACAA2‐K214R plasmid in the MDA‐MB‐231 and MDA‐MB‐468 cell lines and the upregulation and knockdown of LDHC4 expression affect ACAA2 catalytic activity. L) Schematic diagram illustrating how LDHC4 regulates ACAA2 lactylation to alter protease activity, thereby affecting substrate acetyl‐CoA levels. All data are presented as mean ± SD. Student's *t*‐test or the Mann – Whitney test was performed in F–K. RMSD: root mean square deviation, ns, not significant, *p < 0.05, **p < 0.01, ***p < 0.001.

Given that ACAA2 is critical for the final step of both the HADHA and ECHS1 catalytic pathways and the β‐oxidation of FAs (**Figure** **4**A) and because ACAA2 was lactylated to a greater extent (OE/Mock ratio = 6.50, Figure 3H) than HADHA or ECHS1 in both the LDHC4‐Mock and LDHC4‐OE groups (Figure 3H), our research focused on the impact of lactylation on the catalytic activity of ACAA2. To better investigate the impact of K residue lactylation on the catalytic activity of the enzymes, we first determined whether the lactylation sites of ACAA2, are conserved across mammalian species. Sequence alignment of human and mouse ACAA2 revealed that the lactylation sites (K214, K270) in ACAA2 enzymes are located in conserved regions (Figure 4A). Molecular dynamics simulations revealed that K214 lactylation (K214lac) markedly enhanced the global structural stability of ACAA2 while specifically stabilizing its 130–150 domain; the modification exerted a pronounced inhibitory effect on protein conformational contraction (Figure 4B). During the 30‐ns simulation trajectory, the wild‐type ACAA2 attained its lowest‐energy contracted state at frame 29 995, whereas the ACAA2‐K214lac achieved a more stable conformational state by frame 29 988 (Figure 4C). Structural alignment of these minimal‐energy states (RMSD = 0.764 Å) revealed significant secondary structure reorganization in the 120–160 peptide segment and pronounced conformational changes in the modification‐proximal 209–214 region of ACAA2‐K214lac (Figure 4D). These lactylation‐induced structural perturbations are likely to have functionally significant effects on ACAA2 activity mediated by K214lac. We further constructed plasmids bearing wild‐type and mutant (K214R or K270R; K>R [lysine > Arginine]) ACAA2 and confirmed that Cho cells (selected for their low baseline lactylation levels) transfected with these plasmids exhibited increased ACAA2 protein expression (Figure 4E,F). Co‐IP and immunoblotting with a pan‐lactylation antibody revealed a significant decrease in ACAA2 lactylation in the ‐K214R and ‐K270R groups compared with the ‐WT group (Figure 4G). Moreover, there was no significant difference in ACAA2 protein expression levels in the input samples subjected to co‐IP among the three groups, indicating that the K214R and K270R mutations did not affect global ACAA2 protein expression and instead decreased ACAA2 lactylation (Figure 4G).

To determine the catalytic activity of ACAA2 in Cho cells, we measured the levels of the ACAA2 substrate acetyl‐CoA as an indirect reflection of ACAA2 activity. The acetyl‐CoA levels in the ACAA2‐WT, ‐K214R, and ‐K270R groups were significantly lower than those in the ACAA2‐Mock group; the ACAA2‐K214R group yielded higher acetyl‐CoA levels than both ACAA2‐WT and ‐K270R groups, but we observed no difference between the ACAA2‐WT and ‐K270R groups (Figure 4H). To further test the impact of the K214R and K270R mutations on the catalytic activity of ACAA2, we treated cells with sodium L‐lactate to induce lactylation.^[^ ^16^ ^]^ We observed increased ACAA2 lactylation levels in the ACAA2‐WT, ‐K214R, and ‐K270R groups following induction with 10 mM sodium L‐lactate treatment. After lactate induction, ACAA2 lactylation levels were higher, and acetyl‐CoA levels were lower in the ACAA2‐WT and ‐K270R groups compared to the ‐K214R group, with no significant difference observed between the ACAA2‐WT and ‐K270R groups (Figure 4I,J), hinting that K214R might influence the catalytic activity of ACAA2. We then measured the acetyl‐CoA levels in MDA‐MB‐231 and MDA‐MB‐468 cells transfected with ACAA2‐WT, ‐K214R, ‐K270R, LDHC4‐OE, and Si‐LDHC4 and observed results similar to those from the Cho cells, namely, that the acetyl‐CoA levels in the ‐WT and ‐K270R groups were lower than those in the ‐K214R group and similar to one another (Figure 4K). Collectively, LDHC4 overexpression decreased acetyl‐CoA levels, while LDHC4 knockdown increased acetyl‐CoA levels in both MDA‐MB‐231 and MDA‐MB‐468 cells (Figure 4K). These results confirmed that K214lac alters ACAA2 catalytic activity in TNBC cells (measured by acetyl‐CoA levels) and that LDHC4 impacts the catalytic activity of ACAA2 by promoting lactylation (Figure 4L).

### 2.5 Enhanced ACAA2 Activity Promotes MDA‐MB‐231 Cell Growth and Proliferation In Vitro and In Vivo

**Figure 5:**
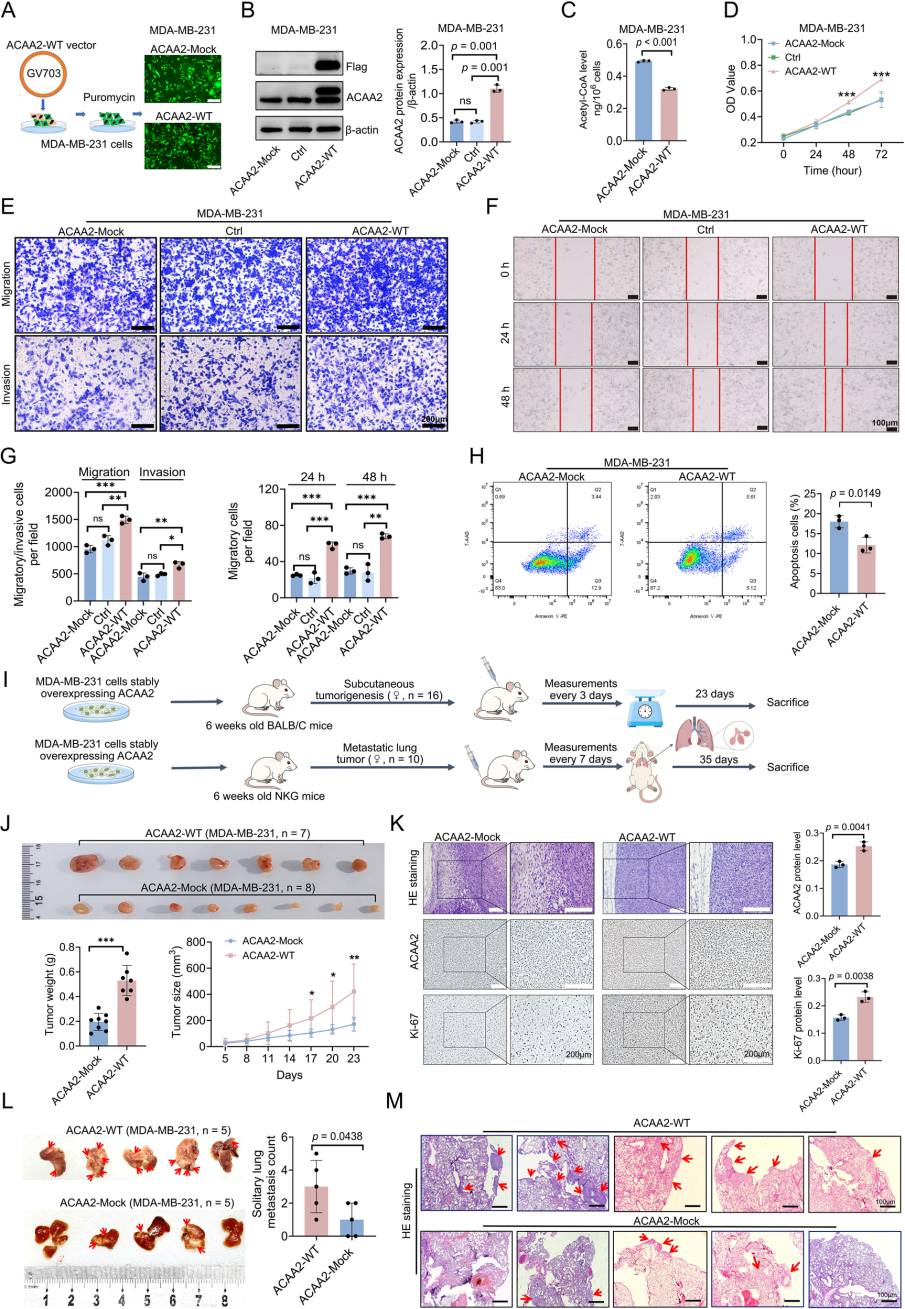
Altered ACAA2 activity affects TNBC progression. A) The establishment of the MDA‐MB‐231 cell line with stable ACAA2 expression. B,C) ACAA2 catalytic activity was stimulated by upregulating ACAA2 expression in MDA‐MB‐231 cells; ACAA2 catalytic activity was reflected by detecting the content of an ACAA2 substrate, acetyl‐CoA. D) Cells with lentivirus‐mediated ACAA2‐WT, ACAA2‐Mock (empty lentivirus vector), and Ctrl (untreated cells) were analysed via CCK‐8 assay. E–G) transwell migration and invasion assays and wound healing analysis. Images were taken at 0 h and 48 h for transwell assays and at 0 h, 24 h, and 48 h for wound healing analysis. H) Flow cytometry (FCM) using Annexin V‐PE/7‐AAD double fluorescent dye was performed to evaluate cell apoptosis. I) Schematic of the mouse subcutaneous tumorigenesis and pulmonary metastasis model. J) Photographs of xenograft tumors (n = 7 or n = 8 mice per group; one mouse in the ACAA2‐WT group was excluded from the study due to post‐inoculation health complications) derived from ACAA2‐WT/‐Mock MDA‐MB‐231 cells. Growth curves of the tumors from the different groups were generated, and the masses of the removed tumors were quantified. K) IHC was used to assess the expression of ACAA2 and Ki‐67 in the tumor tissues. HE staining was also performed to confirm tumor formation. L,M) Lung metastasis imaging and tumor burden quantification were performed in female NKG mice bearing ACAA2‐WT or ACAA2‐Mock tumors (n = 5 per group), with histopathological confirmation via HE staining. The red arrow indicates the tumor. All data are presented as mean ± SD. Student's *t*‐test or the Mann – Whitney test was performed in B, C, D, G, H, J, K, and L. IHC: Immunohistochemistry, ns, not significant, * *p* < 0.05, ** *p* < 0.01, *** *p* < 0.001.

After determining that ACAA2 catalytic activity is affected by ACAA2‐K214lac, we investigated whether ACAA2 activity affects TNBC growth and proliferation. To model enhanced ACAA2 protein activity, we used a lentivirus system to overexpress ACAA2 in MDA‐MB‐231 cells. Following puromycin selection, we established a stable wild‐type (WT) ACAA2‐overexpressing (ACAA2‐WT) MDA‐MB‐231 cell line (**Figure** **5**A,B). Consequently, the catalytic activity of ACAA2 in the ACAA2‐WT group was markedly increased, as reflected by a significant decrease in acetyl‐CoA expression (Figure 5C). We first evaluated the viability of MDA‐MB‐231 cells following ACAA2 overexpression, and the findings suggested that enhanced ACAA2 catalytic activity accelerated cell growth (Figure 5D). Subsequently, we assessed the migration and invasion capabilities of ACAA2‐WT/‐Mock MDA‐MB‐231 cells. The results demonstrated that compared with Ctrl and Mock cells, cells with increased ACAA2 catalytic activity presented significantly greater migration and invasion capabilities (Figure 5E–G). Flow cytometry (FCM) assay confirmed that up‐regulation of ACAA2 catalytic activity reduced apoptosis in MDA‐MB‐231 cells (Figure 5H). To confirm these findings in vivo, xenograft experiments were conducted with ACAA2‐WT MDA‐MB‐231 cells and ‐Mock vector‐transfected cells (Figure 5I), and the tumor volume and weight were monitored; both of these parameters were greater in the ACAA2‐WT group than in the ‐Mock group (Figure 5J). We further assessed ACAA2 and Ki‐67 expression in the ACAA2‐WT/‐Mock mice tissues via IHC analysis, which confirmed that ACAA2 protein expression was substantially increased in the ACAA2‐WT group and was accompanied by increased Ki‐67 expression (Figure 5K). In the NKG mouse model of distant lung metastasis, the ACAA2‐WT group demonstrated a significantly higher number of lung metastatic tumors compared with the ACAA2‐Mock group (Figure 5L,M). These in vitro and in vivo findings suggest that increased ACAA2 activity promotes MDA‐MB‐231 cell progression.

### 2.6 Enhanced ACAA2 Activity Promotes Lipid Metabolism and FFA Production

ACAA2 is as a key catalytic enzyme involved in mitochondrial FA elongation.^[^ ^25^ ^]^ Because we hypothesized that enhanced ACAA2 activity due to LDHC4‐induced lactylation regulates FA metabolism, we conducted targeted lipid and energy metabolism analyses of ACAA2‐OE and ‐Mock transfected MDA‐MB‐231 cells. In the ACAA2‐WT cells compared to the ACAA2‐Mock group cells, 140 lipids were upregulated, and 97 were downregulated (Figure S1A, Supporting Information), with the top 20 differentially abundant lipids predominantly comprising cholesteryl esters, ceramides, and phosphatidic acids (Figure S1B, Supporting Information). KEGG and Human Metabolome Database (HMDB) enrichment analyses of the differentially abundant lipids suggested that ACAA2 may be associated with adipokine signaling, fat digestion and absorption, the metabolism of various lipid subclasses, and metabolic diseases (Figure S1C, D, Supporting Information). Pearson correlation analysis of the significantly differentially abundant lipids to explore their inter‐regulatory relationships during metabolism in TNBC revealed a negative correlation between the intracellular levels of triglyceride and most lipid compounds, including ceramides, phosphatidylethanolamines, and phosphatidylcholines, with increased ACAA2 expression/activity (Figure S1E, Supporting Information). Cluster analysis of targeted energy metabolism profiles was conducted on 55 different metabolites classified into 10 major categories based on their properties. The results showed that nucleotide metabolites and organic acids and their derivatives were significantly higher in the ACAA2 ‐ Mock group than in the ACAA2‐OE group, indicating that enhanced ACAA2 activity may inhibit the metabolism of these compounds (Figure S1F, Supporting Information). Compared with those in the ‐Mock group, the ACAA2‐OE group exhibited greater levels of FFAs, ceramide‐NS (Cer‐NS), and phosphatidylcholine‐PC‐O (PC‐O), whereas the levels of cholesteryl ester (CE), lysophosphatidylcholine (LPC), and hexosylceramide‐NS (HexCer‐NS) were lower in the ACAA2‐OE group than in the ‐Mock group (Figure S1G, Supporting Information). Importantly, the ACAA2 substrate acetyl‐CoA was also found to be decreased in ACAA2‐OE MDA‐MB‐231 cells (Figure S1G, Supporting Information), which is consistent with our previous results (Figure 5C). These findings suggest that increased ACAA2 expression and the subsequent increase in ACAA2 catalytic activity promote the synthesis of FFAs as well as lipids in the Cer‐NS and PC‐O subclasses while inhibiting the synthesis of lipids in the acetyl‐CoA, CE, LPC, and HexCer‐NS subclasses (Figure S1G, Supporting Information).

Lipid function is closely related not only to lipid content but also to chain length (i.e., the total number of carbon atoms in the FA chains).^[^ ^26^ ^]^ Further comparison of lipids with different chain lengths in the ACAA2‐WT and ‐Mock groups revealed that the levels of FFAs, Cer‐NS, and PC‐O with various chain lengths were greater in the ACAA2‐OE group, whereas those of CE, LPC, and HexCer‐NS were lower (Figure S1H, Supporting Information), indicating that increased ACAA2 expression and activity promote the elongation of FA chains in FFAs, Cer‐NS, and PC‐O and inhibit the elongation of FA chains in CE, LPC, and HexCer‐NS. Moreover, targeted energy analysis of metabolic profiles further confirmed that the overexpression of ACAA2 downregulated the expression of fumaric acid, and cis‐aconitic acid, but increased the synthesis of L ‐ alanine, and serine in MDA‐MB‐231 cells (Figure S1I, Supporting Information).

### 2.7 LDHC4 Regulates FFA Production by Modulating ACAA2 Activity through Lactylation

**Figure 6:**
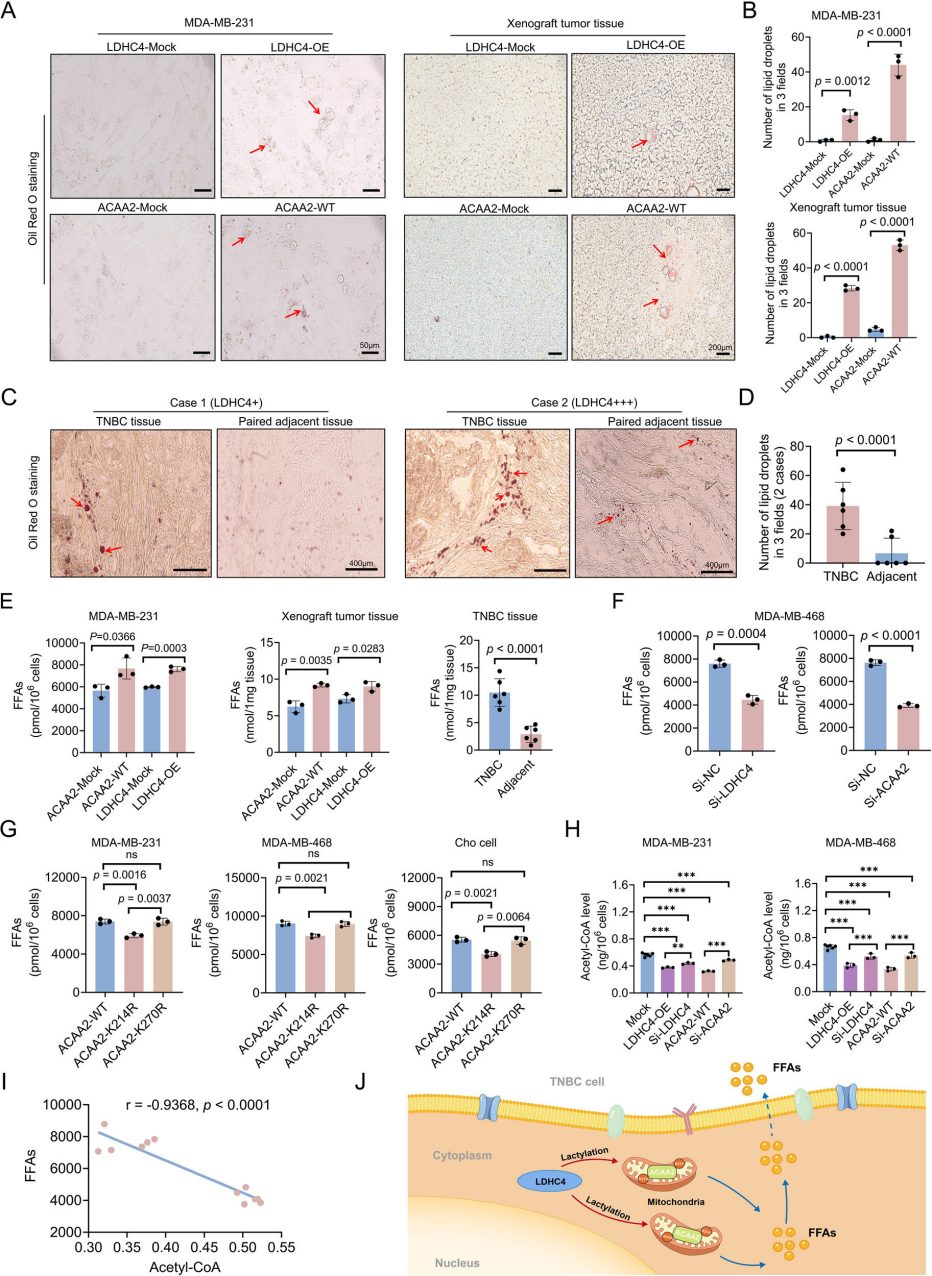
LDHC4 regulates FFA production by promoting the lactylation of ACAA2. A,B) Oil Red O staining revealed increased lipid droplet formation in MDA‐MB‐231 cell lines and mice xenograft tumors with LDHC4‐OE and ACAA2‐WT; both intracellular and extracellular lipid droplets (red arrows) were observed. C,D) Detection of lipid droplets in TNBC and adjacent nontumor tissues using Oil Red O Staining (n = 2). E) Elevated FFA levels in MDA‐MB‐231 cell lines and xenograft tumors with LDHC4‐OE and ACAA2‐OE; FFAs levels in TNBC and adjacent nontumor tissues were measured using a colorimetric/fluorometric assay. F) Knockdown of LDHC4 or ACAA2 reduces FFA levels in the MDA‐MB‐468 cell line. G) Lactylation of ACAA2 at K214 affects FFA levels. H) LDHC4 or ACAA2 upregulation or knockdown affects acetyl‐CoA content in MDA‐MB‐231 and MDA‐MB‐468 cells, indirectly reflecting ACAA2 catalytic activity. I) Negative correlation between acetyl‐CoA content and FFA levels in the MDA‐MB‐231 cell line. J) Schematic illustrating how LDHC4 promotes FFA accumulation by inducing ACAA2 lactylation. All data are presented as the mean ± SD. Student's *t*‐test or the Mann – Whitney test was performed in B, D, E, F, G, and H, and Spearman correlation coefficient analysis was utilized in I. ns, not significant, ** *p* < 0.01, ***p < 0.001.

As our targeted lipid metabolism analysis demonstrated that FFA levels are elevated in ACAA2‐OE MDA‐MB‐231 cells, we further investigated whether LDHC4‐OE or ACAA2‐WT could produce lipid droplets in both MDA‐MB‐231 cells and mouse model tumor tissues. Oil Red O staining of MDA‐MB‐231 cells and tumor tissues from nude mice in the LDHC4‐OE and ACAA2‐WT groups revealed that overexpression of either protein increased the number of lipid droplets (**Figure** **6**A,B). Further analysis of lipid droplet formation in LDHC4‐positive TNBC specimens and paired adjacent nontumor tissues revealed significantly higher lipid droplet accumulation in tumor tissues compared with paired adjacent nontumor controls (Figure 6C,D). Moreover, further assessment of FFA levels revealed that mouse tumor tissues and MDA‐MB‐231 cells from the LDHC4‐OE and ACAA2‐WT groups presented higher FFA levels than those from the corresponding ‐Mock groups (Figure 6E), correspondingly, TNBC tissues with LDHC4 expression exhibited significantly higher FFA levels compared to matched adjacent nontumor tissues (Figure 6E), but LDHC4 and ACAA2 knockdown (with Si‐LDHC4 and Si‐ACAA2, respectively) in MDA‐MB‐468 cells had the opposite effects (Figure 6F). Moreover, FFA content was lower in the ACCA2‐K214R group than in either the ACCA2‐K270R or ACCA2‐WT group in MDA‐MB‐231, MDA‐MB‐468, and Cho cells (Figure 6G). This finding raised the question of whether LDHC4 promotes TNBC progression by modulating ACAA2 activity to regulate FFA production. Further analysis of acetyl‐CoA levels in LDHC4‐ and ACAA2‐overexpressing and knockdown MDA‐MB‐231 or MDA‐MB‐468 cells indicated that intracellular acetyl‐CoA content is influenced by LDHC4 and ACAA2 levels (Figure 6H). Critically, the FFA content was found to be negatively correlated with the level of acetyl‐CoA (Figure 6I). As shown in Figure 3I, LDHC4 overexpression promoted an increase in ACAA2 lactylation both in mouse tumor tissues and MDA‐MB‐231 cells. Additionally, in Cho, MDA‐MB‐231, and MDA‐MB‐468 cells, the lactylation of ACAA2 was associated with increased ACAA2 catalytic activity (Figure 4H,K). Therefore, these results suggest that LDHC4 influences ACAA2 catalytic activity by promoting ACAA2 lactylation (K214lac), subsequently regulating the production of intermediary metabolite FFAs, leading to FFA accumulation (Figure 6J).

### 2.8 FFA Accumulation Promotes TNBC Progression In Vitro and In Vivo

**Figure 7:**
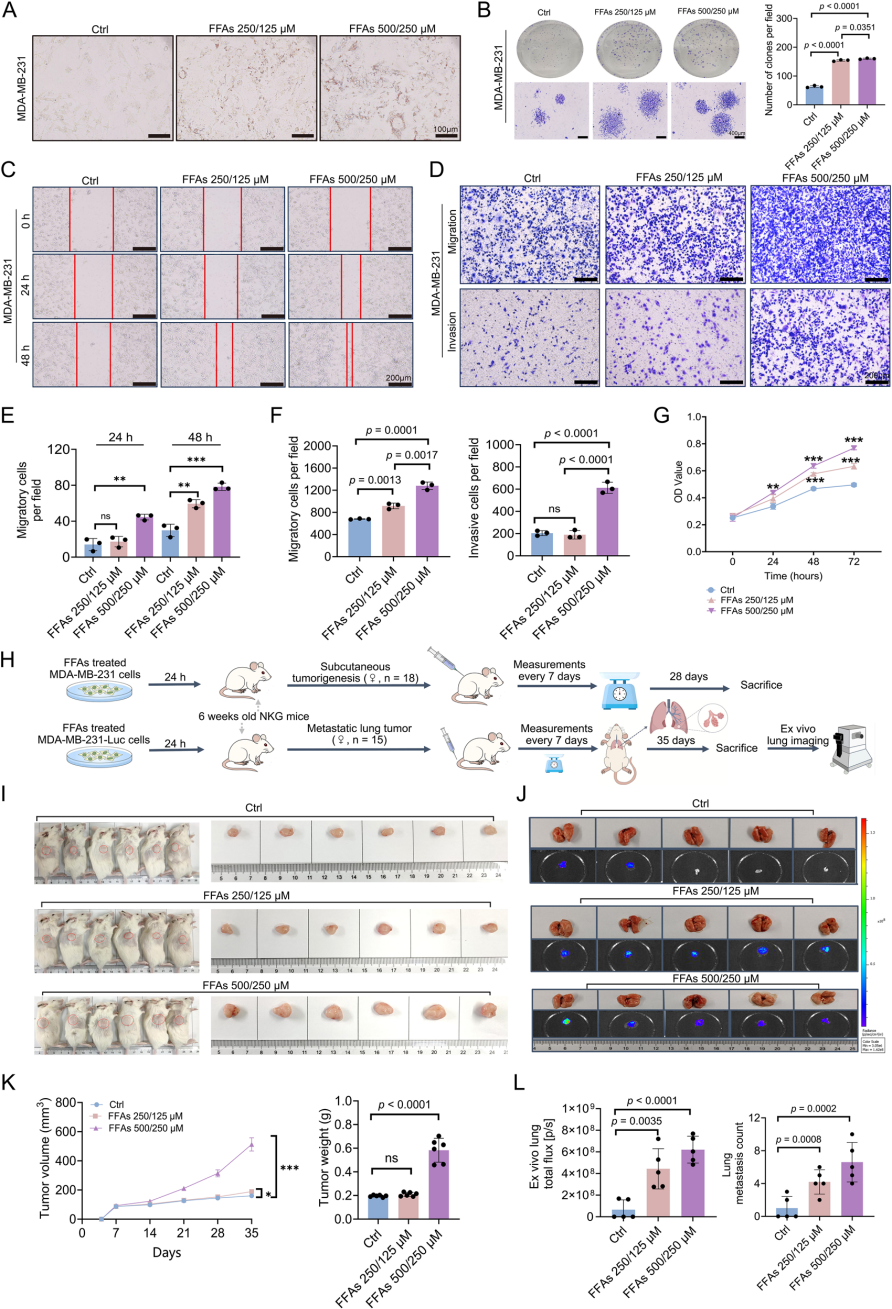
Effects of FFAs on the biological functions of MDA‐MB‐231 cells. A) Oil Red O staining demonstrates lipid droplet formation in MDA‐MB‐231 cells following various FFAs treatments. B) Colony formation assay. C–F) Wound healing and transwell assays were used to assess cell migration and invasion capabilities. Images were taken at 0, 24, and 48 h for the wound healing assay and at 0, and 48 h for the transwell assay. G) The CCK‐8 assay was used to plot growth curves following various FFAs treatments. H) Diagram of tumorigenesis in vivo. I) Subcutaneous tumor development profile in NKG mice (n = 6 per group); red circles demarcate subcutaneous tumor positions. J) Metastatic burden in pulmonary tissues of NKG mice (n = 5 per group). K) The effects of different concentrations of FFAs on subcutaneous tumor formation in NKG mice were evaluated by comparing tumor growth curves and tumor weight across groups. L) Ex vivo analysis of total lung flux and metastatic lesion counts in NKG mice across different FFA concentration groups. The FFAs mixture used for cell coculture was a 2:1 ratio of sodium oleate to sodium palmitate, resulting in final concentrations. All data are presented as mean ± SD. Student's *t*‐test or the Mann – Whitney test was performed in B, E, F, G, K, and L. ns, not significant, *p < 0.05, ** *p* < 0.01, *** *p* < 0.001 versus Ctrl group.

FA/FFAs can rewire metabolic programs in BC cells and influence cell tumorigenicity and aggressiveness.^[^ ^27^ ^]^ Given that FFA accumulation due to an LDHC4‐mediated increase in ACAA2 catalytic activity may ultimately influence cell behaviors, we then investigated the impact of FFAs on the biological functions of MDA‐MB‐231 cells. Oil Red O staining of MDA‐MB‐231 cells treated with 250/125 or 500/250 µm FFAs indicated an increase in the number of lipid droplets with increasing FFA concentration (**Figure** **7**A). Treatment with FFAs was found to enhance the growth, proliferation, clonogenicity, migration, and invasion of MDA‐MB‐231 cells in a concentration‐dependent manner (Figure 7B–G). In parallel, we developed experimental models of both subcutaneous tumorigenesis and pulmonary metastasis in NKG mice (Figure 7H). In vivo tumorigenesis and pulmonary metastasis experiments confirmed that FFAs facilitated the formation of MDA‐MB‐231 cell‐derived tumors in mice in a concentration‐dependent manner, as the tumor volume, weight and metastatic lesions increased as the FFAs concentration increased (Figure 7I–L).

We conducted parallel experiments to inhibit FFAs using the specific inhibitor GLPG0974 (Molecular formula: C_2__5_H_2__5_ClN_2_O_4_S; half maximal inhibitory concentration [IC_50_]: 13.10 nm), which significantly reduced endogenous FFAs levels in MDA‐MB‐231 cells (Figure S2A,B, Supporting Information). In vitro assays demonstrated that FFAs inhibition suppressed cellular proliferation, migration and invasion capabilities (Figure S2C–F, Supporting Information). Furthermore, GLPG0974 treatment markedly attenuated the tumor‐forming capacity of MDA‐MB‐231 cells in NKG mouse xenograft models (Figure S2G–I, Supporting Information). These in vitro and in vivo findings provide counterevidence that FFAs accumulation ultimately promotes TNBC progression.

### 2.9 FFAs Promote TNBC Progression by Activating Autophagy and Regulating the Cell Cycle

**Figure 8:**
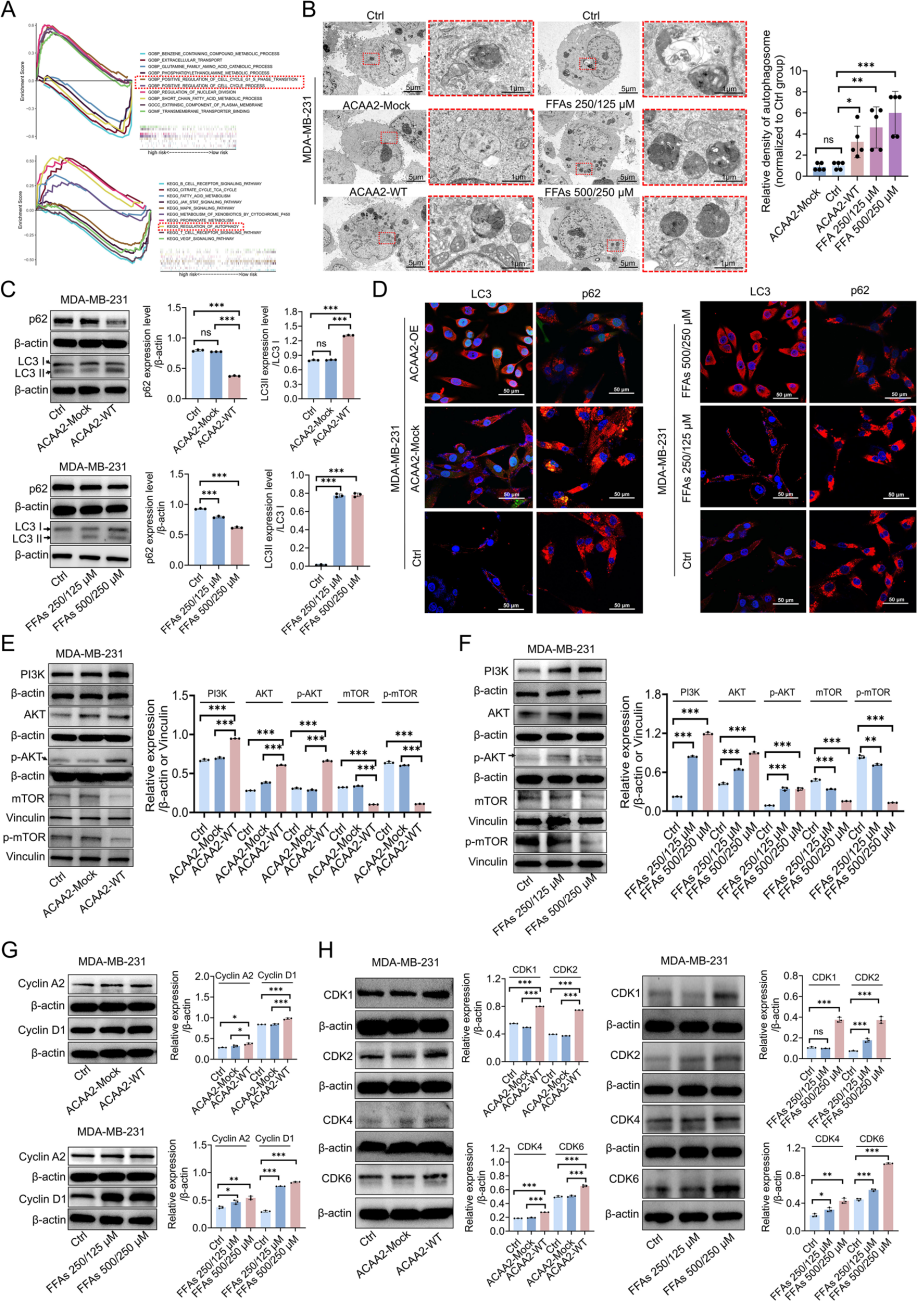
Impact of ACAA2 overexpression and FFAs on the autophagy and cell cycle pathways. A) GSEA suggested that ACAA2 may regulate autophagy and the cell cycle in TNBC (indicated by the red outline). B) Transmission electron microscopy was used to assess autophagosome formation. Relative quantification of autophagosome density was normalized to the Ctrl group. C) Immunoblotting to measure the expression of autophagy related proteins p62 and LC3 in MDA‐MB‐231 cells after either ACAA2 overexpression or FFAs treatment alone. D) Representative indirect immunofluorescence images of p62 and LC3 distribution in MDA‐MB‐231 cells. p62 or LC3 protein was by labelled by red fluorescence Cy3, and nucleus was localized by blue fluorescence 4',6‐diamidino‐2‐phenylindole (DAPI). E,F) PI3K/AKT/mTOR‐mediated autophagy was determined in MDA‐MB‐231 cells by immunoblotting after either ACAA2 overexpression or FFAs treatment alone. G,H) Immunoblotting confirmed the changes in the expression of cell cycle‐related proteins following ACAA2 overexpression or FFAs treatment alone. p62 (Panel C) and AKT (Panel E) share the same reference β‐actin due to being from the same PVDF membrane; Cyclin A2 (Panel G) and CDK2 (Panel H) share β‐actin (same membrane) as well. All data are presented as mean ± SD. Student's *t*‐test or the Mann – Whitney test was performed in B, C, E, F, G, and H. GSEA: gene set enrichment analysis, ns, not significant, * *p* < 0.05, ** *p* < 0.01, *** *p* < 0.001.

Having discovered that FFAs promote TNBC progression, we next aimed to assess the associated mechanism. Using The Cancer Genome Atlas (TCGA) database, we conducted Gene Set Enrichment Analysis (GSEA) to identify ACAA2‐related pathways in TNBC. The GSEA results suggested that ACAA2 is associated with autophagy and cell cycle regulation and can modulate FA metabolism in TNBC (**Figure** **8**A). This prompted us to ask whether FFAs might promote TNBC progression by affecting cellular autophagy and the cell cycle. Given that ACAA2 activity impacts FFA levels, we aimed to address this question by exploring the relationships of ACAA2 (and FFAs) with autophagy and cell cycle regulation. Transmission electron microscopy was performed to verify the induction of autophagy, and the results revealed vacuole‐like structures composed of a double membrane and more transparent structures containing cellular debris (autophagosomes) in the cytoplasm of MDA‐MB‐231 cells, with greater numbers (or increased density) of autophagosomes observed in the ACAA2‐WT and FFA‐supplemented groups (Figure 8B). We then evaluated the expression of autophagy related proteins such as p62, LC3I and LC3II. Immunoblotting of MDA‐MB‐231 cells overexpressing ACAA2 or treated with FFAs revealed that the autophagy substrate p62 was downregulated by both ACAA2 overexpression and FFA treatment, whereas the LC3II/LC3I ratio was increased (Figure 8C), indicating that both ACAA2 overexpression and FFAs supplementation can promote the degradation of p62, accelerate the conversion of LC3I to LC3II, and thus activate early cell autophagy in MDA‐MB‐231 cells. Immunofluorescence staining corroborated the immunoblotting results, demonstrating p62 was decreased and LC3 was upregulated by both ACAA2 overexpression and FFA treatment (Figure 8D). Given that the phosphoinositide 3‐kinase (PI3K)/protein kinase B (AKT)/mammalian target of rapamycin (mTOR) pathway is a critical regulator of autophagy,^[^ ^28^ ^]^ we further determined whether FFAs trigger PI3K/AKT/mTOR‐mediated autophagy. As we predicted, the levels of PI3K, AKT, and p‐AKT were elevated in both the ACAA2‐WT and FFA‐supplemented MDA‐MB‐231 cells, and the levels of mTOR and p‐mTOR were decreased (Figure 8E, F).

Finally, we confirmed the impacts of ACAA2 overexpression and FFAs on the expression levels of cell cycle‐related proteins. Cyclin A2, cyclin D1, CDK1, CDK2, CDK4, and CDK6 were upregulated in the ACAA2‐WT groups, exceeding the levels in the corresponding Mock and Ctrl groups (Figure 8G,H), suggesting that increased ACAA2 expression and activity promote cell cycle progression. As expected, treatment with FFAs also promoted the expression of cell cycle related proteins in MDA‐MB‐231 cells (Figure 8G,H), and this effect was concentration‐dependent; these results were consistent with those in the ACAA2‐WT group. All these results supported our hypothesis that LDHC4 promotes TNBC progression by modulating ACAA2 activity to regulate FFA production, and the accumulation of FFAs further induces autophagy and accelerates the cell cycle, ultimately fostering TNBC progression (graphical abstract).

### 2.10 ACAA2 and ACAA2‐K214lac as Potential Therapeutic Targets for TNBC

**Figure 9:**
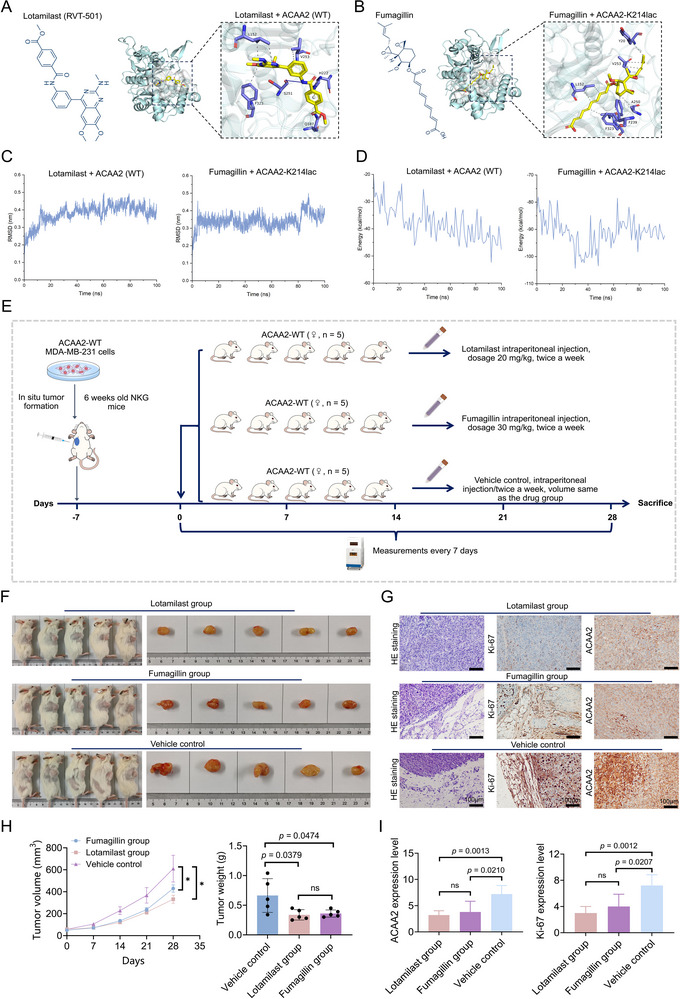
ACAA2 and ACAA2‐K214lac emerge as novel therapeutic targets in TNBC. A,B) Virtual screening via molecular docking revealed Lotamilast as the top‐scoring compound for wild‐type ACAA2, whereas Fumagillin showed preferential binding to ACAA2‐K214lac. C,D) Molecular dynamics simulations (MM‐PBSA binding free energy analysis) assessed the binding interactions of Lotamilast with wild‐type ACAA2 and Fumagillin with ACAA2‐K214lac. E) Therapeutic protocol in animal models. F) Comparative analysis of tumor dimensions among all experimental groups following 28 days of treatment. G) Immunohistochemical analysis assessed Ki‐67 and ACAA2 expression across treatment groups, while HE staining evaluated tumor morphology. H) The therapeutic efficacy of both agents in NKG mice was assessed through comparative analysis of tumor growth curves (Tumor volume–based change) and final tumor weights among treatment groups. I) Immunohistochemical scoring was employed to quantify treatment‐induced alterations in Ki‐67 and ACAA2 expression patterns across treatment groups. Continuous variables are expressed as mean ± SD. Student's *t*‐test or the Mann – Whitney test was performed in H, and I. MM ‐ PBSA: molecular mechanics Poisson ‐ Boltzmann surface area, ns, not significant, * *p* < 0.05 versus Vehicle control.

This study systematically evaluated the potential of ACAA2 and its modified form, ACAA2‐K214lac, as therapeutic targets in TNBC. Through rigorous computational screening combining molecular docking simulations with MM ‐ PBSA binding free energy calculations, we identified Lotamilast as the most potent binder to wild‐type ACAA2, demonstrating superior binding thermodynamics, while Fumagillin emerged as the optimal binder to ACAA2‐K214lac (**Figure** **9**A–D). Based on these structural data, we conducted in vivo studies in NKG mice with orthotopically implanted ACAA2‐positive tumors (Figure 9E). The experimental cohort, comprising 15 tumor‐bearing subjects, underwent randomized allocation into three parallel groups: Lotamilast therapy (20 mg kg^−1^), Fumagillin therapy (30 mg kg^−1^), and Vehicle control, with five biologically independent replicates per group (Figure 9E). Both therapeutic agents were delivered via precisely calibrated intraperitoneal injections administered twice weekly over a continuous 28‐day intervention period (Figure 9E). After 28 days of treatment, both Lotamilast‐ and Fumagillin‐treated groups exhibited significantly reduced tumor volume and weight compared to the vehicle control group, with no statistically significant difference observed between the two treatment groups (Figure 9F,H).  Complementary IHC profiling of harvested tumors provided mechanistic validation, uncovering pronounced suppression of the proliferation marker Ki‐67 alongside substantial attenuation of ACAA2 protein expression in both therapeutic cohorts compared to control specimens (Figure 9G,I). These findings suggest that ACAA2 and ACAA2‐K214lac represent promising therapeutic targets for TNBC.

## 3 Discussion

LDHC4 has previously been highlighted as a targetable CTA for immunotherapy in BC.^[^ ^5^ ^]^ Our study revealed that LDHC4 is overexpressed in TNBC tissues and that high LDHC4 expression is a prognostic factor in TNBC. In addition, LDHC4 promotes TNBC progression both in vitro and in vivo. We then investigated whether lactylation plays a role in the mechanism by which LDHC4 promotes TNBC progression. We found that LDHC4 enhances the lysine lactylation of one of the catalytic enzymes involved in FA metabolism—ACAA2. The increased lactylation of ACAA2, a key enzyme in the final step of FA β‐oxidation, increases its catalytic activity, thereby accelerating FA metabolism. This acceleration in FA metabolism leads to the accumulation of intermediate FFAs, which ultimately promotes TNBC progression by regulating autophagy and cell cycle progression.

As a CTA, LDHC4 has been reported to be elevated in various cancers, and its elevation has prognostic value in patients with these diseases.^[^ ^4^, ^6^, ^7^, ^8^, ^9^, ^10^ ^]^ In LUAD, the proportion of tumor tissues expressing LDHC4 is as high as 81.8%, and higher LDHC4 expression is associated with shorter OS and more advanced TNM stage.^[^ ^6^ ^]^ Our previous findings in LUAD and HCC showed that LDHC4 protein levels are increased in tumor tissues and are negatively associated with patient OS.^[^ ^8^, ^9^ ^]^ In the study of non‐TNBC, we found that LDHC4 protein levels are increased in tumor tissues and shows promise as a prognostic indicator for non‐TNBC; *LDHC* mRNA was detectable in serum and serum‐sourced exosomes and may also be considered a novel biomarker for the diagnosis, treatment efficacy prediction, and recurrence monitoring in non‐TNBC.^[^ ^10^ ^]^ This study strongly confirms the oncogenic role of LDHC4 in TNBC at the cellular, animal model, and patient levels. Specifically, LDHC4 protein expression is increased in TNBC, high LDHC4 expression is an adverse factor that affects TNBC prognosis, and LDHC4 accelerates TNBC cell growth, proliferation, invasion and metastasis. However, only a few studies have focused on the mechanisms of LDHC4 function in such tumors. For example, Chen et al. reported that LDHC4 functions as an oncogene in LUAD, and enhanced LUAD cell growth, proliferation, and tumorigenicity by activating the PI3K/Akt/GSK‐3β pathway.^[^ ^6^ ^]^ In BC, silencing LDHC in four BC cell lines significantly increased the presence of giant cells, nuclear aberrations, DNA damage, and apoptosis.^[^ ^29^ ^]^ Our study is the first to investigate the mechanisms of LDHC4 in TNBC from the aspect of metabolic reprogramming.

The occurrence of metabolic reprogramming in BC is evident,^[^ ^12b^ ^]^ and BC cells exhibit additional metabolic anomalies, such as enhanced lipid synthesis, abnormal amino acid metabolism, and altered lactate metabolism.^[^ ^12^, ^13^, ^14^ ^]^ Protein lactylation occurs when lactate that accumulates during tumor cell metabolism serves as a precursor, inducing histone lysine lactylation.^[^ ^16^, ^18^, ^20^ ^]^ Xiong and colleagues further demonstrated that lactate that accumulates in the tumor microenvironment regulates the N6‐methyladenosine (m6A) modification of tumor‐infiltrating myeloid cells (TIMs) via protein lactylation mediated by the RNA methyltransferase METTL3, enhancing the immunosuppressive function of tumor‐infiltrating myeloid cells and mediating tumor immune evasion.^[^ ^30^ ^]^ On the other hand, lipid metabolic reprogramming has a marked impact on cancer progression by remodeling the tumor microenvironment (TME).^[^ ^31^ ^]^ ACAA2 is a thiolytic enzyme for the final step of FA β‐oxidation, which promotes the metabolism of FAs.^[^ ^32^ ^]^ It has been shown that HCC patients with low ACAA2 levels also tend to exhibit an immunosuppressive microenvironment and are predicted to have a poor prognosis.^[^ ^33^ ^]^ Although the current study was not specifically focused on the TME, we found that lactylation at K214 in ACAA2 influences ACAA2 enzymatic activity, which promotes TNBC progression. Similar studies have shown that protein lactylation plays a crucial regulatory role in the metabolism of HCC cells; for example, lactylation of adenylate kinase 2 (AK2) at K28 significantly impacts its function and subsequent cancer cell behavior.^[^ ^34^ ^]^ Moreover, lactylation of chromobox 3 (CBX3) at K10 mediates its interaction with the epigenetic marker H3K9me3 and facilitates gastrointestinal cancer progression.^[^ ^35^ ^]^ However, the mechanism whereby ACAA2 promotes TNBC progression remains to be disclosed.

Lipid metabolic reprogramming is pronounced in BC cells and relies heavily on both de novo FA synthesis and the uptake of exogenous FFAs from adipocytes for β‐oxidation to meet the high energy demands of BC cells.^[^ ^12^, ^36^ ^]^ The lipidomic analysis in this study revealed that increased ACAA2 expression leads to a substantial increase in FFA levels. Notably, we also found that increased LDHC4 or ACAA2 expression promotes FFA production in MDA‐MB‐231 cells and tumor growth in mouse models. These results suggest that FFAs are involved in the mechanism by which LDHC4 promotes TNBC progression via promoting ACAA2 lactylation. FFAs are vital for energy production,^[^ ^37^ ^]^ and it is therefore unsurprising that many cancer cells show increased dependence on FFA biosynthesis and/or exogenous uptake; this offers an opportunity to counteract cancer progression.^[^ ^38^ ^]^ In this study, we further characterized the functional role of FFAs in TNBC, and found that FFAs accelerate TNBC cell growth and proliferation in a dose‐dependent manner both in vivo and in vitro. Our findings support FFA accumulation as the mediator of LDHC4‐induced ACAA2 lactylation in promoting TNBC progression, and thus the specific mechanism underlying this activity need to be revealed.

It has been demonstrated that FFAs are essential for the activation of intracellular signaling,^[^ ^39^ ^]^ and recent studies have highlighted the association of FAs with autophagy.^[^ ^40^ ^]^ Autophagy is a fundamental form of programmed cell death that occurs across many types of eukaryotic cells.^[^ ^41^ ^]^ Variations in autophagic activity are closely related to BC occurrence and development.^[^ ^42^ ^]^ One study has documented cellular autophagy as an essential survival mechanism through which TNBC cells obtain metabolic substrates under nutrient‐deprivation conditions.^[^ ^43^ ^]^ In our study, we ultimately determined that FFAs induced cellular autophagy in TNBC both in vitro and vivo, connecting FFAs and autophagy in TNBC progression for the first time.

The cell cycle, a complex process regulated by various cyclins and cyclin‐dependent kinases (CDKs), plays crucial roles in cell growth, DNA replication, and cell division, and regulatory disruptions in cell cycle progression have been implicated in the development of nearly all cancers.^[^ ^44^ ^]^ Of the two subtypes of cyclin A, the ubiquitously expressed cyclin A2 and the testis‐specific cyclin A1 areoften found to be upregulated in many tumors.^[^ ^45^ ^]^ Cyclin A2 activates CDK1 and CDK2 to form a complex that plays key roles in controlling the G1/S and G2/M transitions of the cell cycle.^[^ ^46^ ^]^ Like cyclin A2, cyclin D1 can bind CDK4 or CDK6, which regulates the G1/S transition and initiates DNA replication.^[^ ^47^ ^]^ Our results in MDA‐MB‐231 cells suggest that, given the regulation of FFA production by ACAA2, the activity of this enzyme may primarily regulate the cell cycle by controlling FFA accumulation. Thus, we report a new mechanism that could explain the role of LDHC4 in promoting TNBC progression: the overexpression of LDHC4 in TNBC cells enhances ACAA2 lactylation, driving increased ACAA2 enzymatic activity that accelerates FA metabolism, promoting the generation and accumulation of FFAs, which in turn induce autophagy and regulate the cell cycle in TNBC cells, ultimately promoting TNBC progression.

The therapeutic landscape for BC continues to evolve, with emerging strategies combining immune checkpoint inhibitors and cytotoxic agents demonstrating synergistic effects.^[^ ^48^ ^]^ Parallel developments in nanomedicine have yielded novel delivery platforms, including pH‐sensitive siRNA carriers targeting POLR2A,^[^ ^49^ ^]^ advanced nanodelivery systems,^[^ ^50^ ^]^ and photodynamic‐chemotherapy conjugates^[^ ^51^ ^]^ that improve tumor localization while potentiating immune responses. Protein lactylation has recently emerged as a promising therapeutic target in oncology. A review study reported that lactylation modifications regulate both histone and non‐histone proteins, influencing tumor metabolism, immune evasion, and drug resistance.^[^ ^52^ ^]^ Small molecules like tubuloside A inhibit HCC progression by blocking ABCF1 (ATP binding cassette subfamily F member 1)‐K430la modification,^[^ ^53^ ^]^ while LDHA or MCT1 (Monocarboxylate transporter 1) inhibition reduced lactylation levels and reversed chemotherapy resistance.^[^ ^52^, ^54^ ^]^ These findings establish lactylation as a viable target for precision therapy. Our computational studies identified Lotamilast as the optimal binder for wild‐type ACAA2 and Fumagillin as highly selective for the ACAA2‐K214lac. In ACAA2 orthotopic tumor models, both compounds significantly suppressed tumor growth versus controls. Beyond validating ACAA2 as a druggable target, our preclinical studies identify ACAA2‐K214lac as a molecular determinant of inhibitor specificity. This finding enables targeted therapy development for cancers with specific lactylation sites.

Our study has several limitations. First, a significant limitation of research in this field is the current inability to mimic lactylation modifications at specific sites both in vitro and in vivo. Developing methods to add lactate groups to specific protein sites in cellular or animal models remains a critical challenge in ongoing research. Second, we used ACAA2 overexpression as a proxy for enhanced ACAA2 enzymatic activity, and incubation with lactate may lead to the lactylation of other protein sites. Third, this study did not conduct an in‐depth investigation of metabolic differences in LDHC4 expression across BC subtypes. Lastly, the exogenous FFAs used in this study comprised only a mixture of the two predominant FFA species and therefore may not fully mimic the complex FFA composition present in cellular or tumor microenvironments. Consequently, these findings warrant verification through more rigorous investigations in future studies.

## 4 Conclusion

This study comprehensively and systematically examined and determined the underlying mechanisms by which LDHC4 influences FA metabolism to promote TNBC progression via lactylation. Elevated LDHC4 expression drives ACAA2 lactylation at K214, enhancing FA metabolism and generating excess FFAs that activate autophagy and cell cycle progression. LDHC4 may serve as a potential biomarker for TNBC, while the ACAA2‐lactylation axis emerges as a promising novel target for TNBC treatment. The identified mechanism may offer new insights into diagnostic and therapeutic strategies that target lipid metabolism and lactylation to inhibit tumor growth.

## 5 Experimental Section

### Human Samples

This study enroled 99 triple‐negative breast cancer (TNBC) patients receiving treatment at the Fujian Cancer Hospital from March 1, 2018, to June 30, 2023, and matched non‐cancerous controls for each patient. The cohort consisted exclusively of women, and the average patient age was 56 years. Histopathological confirmation was obtained for each case, identifying diverse histological types such as invasive ductal carcinoma and unspecified invasive carcinomas, all lacking ER, PR, and HER2 expression. Monitoring continued until August 31, 2023. Ethical approval was granted by the Ethics Committee of Fujian Cancer Hospital (approval number: SQ2018‐015‐01), and study was carried out according to the Declaration of Helsinki. Informed consent was obtained from the study subjects before their enrolment. The study also included 150 TNBC and 30 adjacent non‐tumor tissue specimens, organized into a commercial high‐throughput TNBC tissue microarray (catalogue no. HBreD180Bc01‐1). All diagnoses were confirmed histopathologically. The specimens were sourced from female patients averaging 52 years in age, covering several histological types, including 42 cases of invasive ductal carcinoma, 96 cases of unspecified invasive carcinoma, 4 cases of medullary carcinoma, 3 cases of metaplastic carcinoma, and 1 case each of mixed metaplastic, basal‐like, squamous cell, adenoid cystic, and mucinous carcinomas. Tissue samples were procured without any intervention, and ethical approval and informed consent were secured prior to collection.

### Main Reagents

The EliVisionTM Plus Two‐Step Detection Kit (Product no. KIT‐9903) was obtained from Fuzhou Maixin (China). LDHC4‐OE, ACAA2‐OE, Si‐LDHC4, and Si‐NC (negative sequence control vector) lentivirus vectors were constructed by GENECHEM (Shanghai, China). The SDS‐PAGE Gel Kit, BCA Protein Quantification Kit were from EpiZyme (Shanghai, China). ECL AB Colour Development Solution Kit (chemiluminescence) was from Meilunbio (Dalian, China). Cell Counting Kit‐8, Oil Red O Staining Kit, and Immunol Fluorence Staining Kit were sourced from Beyotime Biotechnology (Shanghai, China). Annexin V‐PE/7‐AAD Apoptosis Detection Kit was purchased from Sino Biological Inc. (Beijing, China). Acetyl‐CoA Detection Kit was from Nanjing Jiancheng Bioengineering Institute Co., LTD.

### Immunohistochemistry (IHC)

Each tissue sample (TNBC patient sample or mouse tumor model tissue) was embedded in paraffin. The EliVision Plus Two‐Step Detection Kit (Product no. KIT‐9903, Fuzhou Maixin) was used for IHC as specified in the kit manual. Staining was performed with a rabbit monoclonal antibody (mAb) against human LDHC (catalogue no. Ab52747; Abcam, USA) at a dilution of 1:100, a rabbit mAb against human Ki‐67 (catalogue no. Ab16667; Abcam, USA) at a dilution of 1:150, or a mouse mAb against human ACAA2 (catalogue no. sc‐100847, Santa Cruz Biotechnology) at a dilution of 1:100. Phosphate buffered saline (PBS)L was utilized as the negative control. The staining intensity and percentage of positive staining in each section were independently assessed by two pathologists via the double‐blind method. The scoring criteria were as described in our previously published studies:^[^ ^55^ ^]^ final protein expression score = intensity score × percentage score.

### Cell Culture and Groups

Human Cho, MDA‐MB‐231, MDA‐MB‐468, MDA‐MB‐453, BT‐20, HCC1937, and MCF‐7 cells were maintained in high‐glucose DMEM (Gibco, USA), or RPMI‐1640 (Gibco, USA) supplemented with 10% fetal bovine serum (FBS) (Gibco, USA) and 1% penicillin–streptomycin (Gibco, USA) and cultured at 37 °C in 5% CO_2_. All BC cell lines were acquired from the American Tissue Culture Collection (ATCC) or Cell Bank/Stem Cell Bank, Chinese Academy of Sciences. Cho cell line was purchased from Wuhan Pricella Biotechnology Co., Ltd. All cell lines were authenticated by STR profiling. MDA‐MB‐231 and MDA‐MB‐468 cells infected with blank lentivirus vectors, or transfected with blank plasmids were defined as the Mock groups, and cells without any treatment were defined as Ctrl groups.

### Immunoblotting

For other immunoblotting experiments, RIPA lysis solution (Beyotime, Shanghai, China) was used to extract total cellular protein, which was quantified with the BCA Protein Quantification Kit (EpiZyme, Shanghai, China) according to the provided protocol. The target proteins were separated by SDS–PAGE and transferred to a PVDF membrane at a constant voltage of 110 V. The membrane was blocked with 5% non‐fat milk for 1 h and then incubated with rabbit anti‐human LDHC monoclonal antibody (mAb) (Abcam, ab52747, 1:500), mouse anti‐human ACAA2 mAb (Santa Cruz Biotechnology, sc‐100847, 1:500), rabbit anti‐human L‐Lactyl Lysine mAb (PTMBIO, PTM‐1401RM, 1:500), rabbit anti‐human HADHA mAb (Abcam, ab200652, 1:800), rabbit anti‐human Vinculin mAb (Abcam, ab129002, 1:1000), rabbit anti‐human ECHS1 polyclonal antibody (pAb) (Abcam, ab228631, 1:500), mouse anti‐human Flag mAb (Beyotime, AF519, 1:1000), rabbit anti‐human cyclin A2 mAb (Beyotime, AF2524, 1:500), rabbit anti‐human cyclin D1 mAb (Beyotime, AF1183, 1:500), rabbit anti‐human CDK1 mAb (Beyotime, AF1516, 1:800), rabbit anti‐human CDK2 mAb (Beyotime, AG1561, 1:800), rabbit anti‐human CDK4 mAb (Beyotime, AF2515, 1:1000), rabbit anti‐human CDK6 mAb (Beyotime, AF2536, 1:1000), rabbit anti‐human PI3K pAb (Proteintech, 20584‐1‐AP, 1:1000), rabbit anti‐human AKT mAb (Signalway Antibody, 48888, 1:1000), rabbit anti‐human p‐AKT mAb (Thr308) (Cell Signaling Technology, 13038T, 1:1000), mouse anti‐human mTOR mAb (Proteintech, 66888‐1‐Ig , 1:20000), mouse anti‐human p‐mTOR mAb (Ser2448) (Proteintech, 67778‐1‐Ig, 1:5000), rabbit anti‐human LC3 pAb (Sigma‐Aldrich, L8918, 1:300), rabbit anti‐human p62 pAb (Servicebio, GB11531, 1:500), or mouse anti‐human β‐actin mAb (Abcam, ab6276, 1:5000) for 4 h at room temperature or overnight at 4 °C. After washing the membrane three times with TBST, HRP‐labelled sheep anti‐rabbit secondary antibody (ZSGB‐BIO, Beijing, China, 1:10000) or sheep anti‐mouse secondary antibody (ZSGB‐BIO, Beijing, China, 1:10000) was added, and the blots were incubated for 1 h. ECL AB colour development solution (chemiluminescence; Meilunbio, Dalian, China) was added, and the membranes were exposed using a BIO‐RAD ChemiDoc Imaging System. β‐Actin or Vinculin served as an internal reference, and Image J software was employed to quantify expression of the mentioned proteins.

### CCK‐8 Assay

MDA‐MB‐231 and MDA‐MB‐468 cells in the logarithmic growth phase were seeded in a 96‐well plate at a density of 5 × 10^3^ cells per well in a volume of 100 µL per well. The experimental groups included overexpression groups (LDHC4‐OE, and Mock; Genechem, Shanghai, China) and shRNA groups (Si‐LDHC4, and Si‐NC; Genechem, Shanghai, China), with 5 replicate wells per group. After 24, 48, 72, or 96 h of culture at 37 °C in a 5% CO_2_ atmosphere, 10 µL of CCK‐8 reagent (Beyotime, Shanghai, China) was added to each well, and the samples were incubated for an additional 3 h. A spectrophotometer (Multiskan, Thermo) was used to measure the optical density (OD) at 450 and 630 nm. The experiment was repeated three times, and the data were plotted with OD values on the *y*‐axis and time on the *x*‐axis to obtain cell growth curves. The CCK‐8 assay related to ACAA2 and FFAs was conducted following the aforementioned protocol.

### Plate Colony Formation Assay

Stably transfected MDA‐MB‐231 or MDA‐MB‐468 cells in the logarithmic growth phase in monolayers were digested with 0.25% trypsin and dispersed into single cells. The cells were seeded at a density gradient of 50, 100, and 200 cells per dish in 10 mL of prewarmed culture medium at 37 °C and then cultured at 37 °C with 5% CO_2_ and saturated humidity for 2 to 3 weeks with no disturbances. When visible colonies appeared on the dishes, the supernatant was discarded, and the cells were washed twice with PBS. The samples were fixed with 5 mL of pure methanol for 15 min and stained with Giemsa for 10–30 min, after which the colonies were counted.

### Scratch Assay

TNBC cells in the logarithmic growth phase were seeded in a 6‐well plate. A 10 µL pipette tip was used to scratch the BC cell monolayer, and the cells were subsequently incubated for 24–48 h. The width of each scratch was observed under a microscope (Olympus IX73, Japan) at 0, 24, and 48 h. Cells were incubated in incomplete medium (lacking FBS), and those without treatment served as the Ctrl group. The rates at which the scratches healed were determined by counting and averaging the number of cells in three randomly selected fields of view under high magnification and then compared between groups.

### Transwell Assay

Serum‐free medium (300 µL) and Matrigel (60 µL) (Corning, USA) were mixed well at 4 °C, and 100 µL of the mixture was added to the upper chamber of each of three wells and subsequently incubated at 37 °C for 4–5 h. The BC cells were digested with trypsin (Gibco, USA), washed three times with serum‐free medium, counted, and used to prepare a cell suspension. The Matrigel was washed once with serum‐free medium, and 100 µL of the cell suspension was added to each well. Next, 500 µL of medium containing 20% FBS (Gibco, USA) was added to the lower chamber. The transwell chambers were incubated in a 37 °C incubator for 20–24 h. Then, the transwell membranes were removed, washed twice with PBS (HyClone, USA), and fixed with 5% glutaraldehyde at 4 °C. The cells on the membrane were stained with 0.1% crystal violet or Giemsa for 5–10 min at room temperature and washed twice with PBS; any cells remaining on the upper surface of the membrane were removed with a cotton ball. The cells in at least three random fields were counted under a microscope (Olympus IX73, Japan).

### Establishment of Subcutaneous, Orthotopic, and Metastatic Tumor Models in Mice with In Vivo Imaging

Six‐week‐old female BALB/c nude mice (Shanghai SLAC Laboratory Animal Co. Ltd.) were subcutaneously injected with 100 µL of MDA‐MB‐231 or MDA‐MB‐468 cell suspension (3 × 10⁶ cells in saline) into the right upper limb. Tumor size (volume = π/6 × L × W^2^) and body weight were measured every three days. Mice were euthanized via 2% pentobarbital sodium overdose (with pedal reflex confirmation) followed by cervical dislocation. The procedure was performed when tumors reached 20 mm diameter or at 23‐38 days post‐injection (whichever came first). Excised tumors were photographed for analysis. For bioluminescence imaging, mice received intraperitoneal D‐luciferin (10 µL g^−1^, 15 mg mL^−1^), were anesthetized after 20 min, and imaged. Total radiant efficiency quantified fluorescence intensity, correlating with tumor burden. To establish subcutaneous tumor models in NKG mice, MDA‐MB‐231 cells were cultured in DMEM medium containing 10% FBS under standard conditions (37 °C, 5% CO_2_). For subcutaneous implantation, log‐phase cells were trypsinized, washed with PBS, and resuspended at 5 × 10⁷ cells mL^−1^ before combining 1:1 with ice‐cold Matrigel. A 200 µL suspension was injected subcutaneously into the right scapular region of 6–8‐week‐old female mice using a 1 cm needle depth to minimize leakage. Tumor growth was monitored by weekly as described above. For orthotopic models, luciferase‐tagged cells (e.g., MDA‐MB‐231‐Luc, 1 × 10⁷ cells in 200 µL PBS) were delivered to tissue‐specific sites such as the mammary fat pad, with progression quantified by serial bioluminescence imaging. To evaluate hematogenous dissemination, 5 × 10⁵ cells in 100 µL PBS were infused via tail vein after pre‐warming mice at 50°C for vascular dilation, using a 27‐gauge needle for controlled delivery. Metastatic burden in target organs (e.g., lungs) was longitudinally analyzed via in vivo imaging. All the above experiments adhered strictly to the guidelines in the National Institutes of Health *Guide for the Care and Use of Laboratory Animals*. All mice were provided humane care, and the study protocol received approval from the Animal Ethics Center of Fujian Medical University (ethics number: IACUC FJMU2023‐Y‐0131), the Animal Ethics Committee of Shanghai Genechem Co., Ltd (ethics number: GSZE0167404), and the Laboratory Animal Welfare and Ethics Committee of Cyagen Biosciences (Suzhou) Co., Ltd (ethics number: TACU25‐CY014).

### Hematoxylin and Eosin (H&E) Staining

Tissue sections were deparaffinized through sequential xylene washes (xylene I and II, 5–10 min each), rehydrated in graded ethanol (100%, 95%, and 75%, 1–5 min each), and rinsed with water. Nuclei were stained with hematoxylin (5–20 min), differentiated in 1% acid alcohol, and blued in tap water (5 min). Cytoplasm was counterstained with eosin, followed by ethanol dehydration (75–85% for 30 sec, 95% twice, and 100% twice, 0.5–5 min each). The samples were then observed and photographed under a microscope (Olympus IX73, Japan).

### 4D Label‐Free Lactylproteome Expression Analysis (4D‐LFQP‐LA) and Bioinformatics Analysis

4D‐LFQP‐LA and bioinformatics analyses were supported by PTM BIO, Hangzhou, China. Detailed protocols were documented in the previous study.^[^ ^23^ ^]^

### Co‐IP Analysis

Magnetic beads (50 µL; EpiZyme) were washed thrice with 400 µL TBST using magnetic separation. Primary antibodies (ACAA2, 1:30; HADHA, 1:30; ECHS1, 1:50) were diluted in TBST, incubated with beads at 4 °C for 4 h with rotation, then washed four times with PBST. Protein lysates (1 mg) were incubated overnight with antibody‐bound beads at 4 °C. After magnetic separation, complexes were washed four times with PBST, resuspended in RIPA buffer containing 5 × loading buffer (48 µL:12 µL), and boiled at 100 °C for 10 min. Immunoblotting was performed using Anti‐L‐Lactyl Lysine Rabbit mAb (1:500, PTM‐1401RM, PTMBIO, Hangzhou, China).

### Molecular Dynamics Simulation of Lactylated ACAA2‐K214

The ACAA2‐K214 lactylation model was built in PyMOL, and the resulting structure was subsequently optimized at the B3LYP/6‐31G level of theory using the Gaussian 09 software package. Subsequently, electrostatic potential (ESP) calculations were conducted using the B3LYP/6‐311G (d, p) method. All molecular dynamics simulations were performed using GROMACS 2021 with the AMBER99SB‐ILDN all‐atom force field. Long‐range electrostatic interactions were computed using the Particle Mesh Ewald (PME) method. Temperature and pressure were maintained using the V‐rescale thermostat and Parrinello‐Rahman barostat, respectively. The simulation system was neutralized by adding counter‐ions to achieve physiological ionic strength (0.145 M). Energy minimization was initially performed using the steepest descent algorithm for 5000 steps with harmonic restraints applied at a force constant of 2.0 kcal/(mol·Å²). Subsequently, a 100 ps NVT (isothermal‐isochoric) equilibration was conducted with reduced positional restraint of 1.0 kcal/(mol·Å²) to raise the system temperature to 300 K, followed by a 100 ps NPT (isothermal‐isobaric) equilibration at a pressure of one atmospheric pressure. Both temperature and pressure coupling time constants were set to 0.2 ps. Production molecular dynamics simulation was then performed for 30 ns without positional restraints. The integration timestep was 2 fs, and trajectory snapshots were saved every 2 ps for post‐simulation analysis. Non‐bonded interactions were computed using a cutoff of 8.0 Å. All covalent bonds involving hydrogen atoms were constrained using the SHAKE algorithm. Root‐mean‐square deviation (RMSD) and radius of gyration (Rg) analyses were performed using trajectory data recorded at 1 ps intervals to assess system stability. Representative structures from the equilibrated simulation phase were selected for visualization and structural analysis.

### Construction and Transfection of Wild‐Type and Mutant Plasmids

Wild‐type and mutant (K214R, K270R; K>R) *ACAA2* gene sequences were synthesized, cloned and inserted into the pcDNA3.1(+) eukaryotic expression plasmid and then transfected into *E. coli* Top10. Positive clones and new mutant plasmids were synthesized and constructed by Shanghai Shangya Biotechnology Co., Ltd., and verified by sequencing and gel electrophoresis. One day before transfection, the cells were seeded in a 6‐well plate and then transfected once they reached 60% confluence. Two micrograms of plasmid DNA was diluted in 100 µL of OPTI‐MEM (Gibco, USA) and gently mixed. Immediately thereafter, 6 µL of PEI MW 40000 transfection reagent (Yesen, Shanghai, China) was added to 100 µL of diluted DNA, vortexed for 10 s, and thoroughly mixed. The mixture was incubated at room temperature for 10–15 min to form a DNA‒PEI cationic nucleic acid transfection agent mixture. The old growth medium was subsequently removed from the cells, and 2 mL of fresh, prewarmed complete medium was added to each well. One hundred microliters of the DNA‐PEI complex mixture was added to the cells, and the culture plate was shaken gently and then further incubated at 37 °C.

### Detection of Acetyl‐CoA Content

Log‐phase BC cells were prepared in a suspension, and 6 × 10^5^ cells were seeded per well in 6‐well plates (with five replicates per group) and cultured for 24 h under standard conditions. Cells were washed twice with PBS and then cultured in serum‐free medium. Blank wells contained no cells. The plates were incubated at 37°C with 5% CO_2_ for 24 h. Cell supernatants were collected for acetyl‐CoA analysis using an acetyl‐CoA assay kit. The reaction system was prepared according to the kit instructions, with blank wells containing 50 µL of biotin antigen working solution per well, standard wells containing 50 µL of standard solution per well, and test wells containing 50 µL of sample. Biotinylated HRP (not added to blank wells), chromogens A and B, and stop solution were added at 50 µL each. The absorbance OD value was measured at 450 nm using an ELISA (enzyme‐linked immunosorbent assay) plate reader (Thermo) zeroed against blank wells. Measurements were taken within 10 min of adding the stop solution, and the regression equation of the standard curve was used to calculate the acetyl‐CoA content of the samples.

### Flow Cytometry to Detect Cell Apoptosis

Cells from various groups in the logarithmic growth phase were collected, washed twice with precooled (4 °C) PBS, and centrifuged (at 4 °C and 1500 rpm for 5 min) to remove the supernatant, after which the cells were resuspended in 1 × binding buffer to a density of 0.1 × 10^7^ to 1 × 10^7^ cells mL^−1^. The cell suspension was transferred to flow cytometry tubes (100 µL per tube). Unstained cells served as the negative control, and cells stained with either Annexin V‐PE (without 7‐AAD) or 7‐AAD (without Annexin V‐PE) were used to set the flow cytometer voltage and compensation. Untreated experimental control cells were used to determine the baseline levels of apoptotic and dead cells. For all experimental cells, each sample was stained with Annexin V‐PE/7‐AAD at room temperature in the dark for 15 min. Then, 400 µL of 1 × binding buffer was added to stop the reaction, and the cells were analyzed (Beckman Coulter) within one hour.

### Targeted Lipid Metabolomics Analysis and Detection of FFA Content

Cell pellets were lysed in 100 µL ultrapure water containing protease inhibitors (PMSF/EDTA). For lipid extraction, 50 µL suspension was mixed with 500 µL extraction solvent (with internal standards), vortexed (2 min), sonicated (5 min), then supplemented with 100 µL water, vortexed (1 min, 1200 rpm), and incubated (4 °C, 10 min). After centrifugation, 200 µL supernatant was redissolved in resuspension buffer for LC‐MS/MS (Liquid Chromatography‐Mass Spectrometry/Mass Spectrometry). The remaining 50 µL underwent freeze‐thaw cycles (liquid nitrogen) and centrifugation (12 000 rpm, 10 min); supernatant protein concentrations were determined via BCA assay (EpiZyme). UPLC (Ultra Performanxe Liquid Chromatography)‐MS/MS analysis utilized a Thermo Accucore C30 column (2.6 µm, 2.1 × 100 mm i.d.) with gradient elution: mobile phase A (acetonitrile/water with 0.1% formic acid/10 mM ammonium format) and B (acetonitrile/isopropanol with same additives); 0.35 mL min^−1^ flow rate, 45 °C. Electrospray Ionization (ESI) conditions: 500°C, ±5500/4500 V polarity, GS1/GS2 45/55 psi, Curtain Gas (CUR) 35 psi. Cellular FFAs were detected based on UPLC‐MS/MS as detailed above. These experiments and bioinformatics analysis were technically supported by Wuhan Metware Metabolic Biotechnology Co., LTD.  FFAs in TNBC and adjacent nontumor tissues were quantified using the Free Fatty Acid Assay Kit (Quantification, ab65341) following manufacturer's protocols.

### Targeted Energy Metabolism Profiling

TNBC cells were thawed on ice and processed at 4 °C throughout. Cell pellets (100 µL ultrapure water suspension) were extracted with 200 µL chilled methanol (–20 °C), vortexed (2500 rpm, 2 min), and subjected to freeze‐thaw cycles (liquid nitrogen/ice, 5 min each) with repeated vortexing. After centrifugation (12,000 rpm, 10 min, 4 °C), 200 µL supernatant was held at –20 °C for 30 min and recentrifuged. Protein content was determined via BCA assay on reserved aliquots. LC‐MS/MS analysis employed a ACQUITY UPLC BEH Amide column (1.7 µm, 2.1 × 100 mm i.d) with gradient elution: mobile phase A (10 mm ammonium acetate/0.3% ammonia in water) and B (90% acetonitrile/water, v/v) at 0.40 mL min^−1^ (40 °C). The 15‐min gradient transitioned from 5:95 to 30:70, then to 50:50, and finally back to 5:95 A:B (v/v). Electrospray Ionization ESI conditions included: 550°C ionization temperature, voltage ‐4500 V, and CUR 35 psi.

### Oil Red O Staining

The modified Oil Red O Staining Kit (Beyotime, Shanghai, China) was utilized according to the manufacturer's instructions. Briefly, the culture medium was gently removed, the cells were washed once with PBS, 4% paraformaldehyde was added to fix the cells for 10 min, and the cells were washed twice with PBS. Prepared frozen sections stored at –20 °C were allowed to equilibrate on a slide warmer for 5–10 min. Then, 2 mL staining wash solution was added to cover the cells (or frozen sections) for 20 s and then removed, after which 1 mL modified Oil Red O stain was added and the samples were stained for 10–20 min. Subsequently, 2 mL staining wash solution was added for 30 s and then removed, the samples were washed with PBS for 20 s, and the samples were observed and photographed under a microscope (Olympus IX73, Japan).

### Coculture with FFAs

The FFAs mixture used for cell coculture was a 2:1 ratio of sodium oleate and sodium palmitate. MDA‐MB‐231 cells were cultured with 0, 125, 250, or 500 µm of this mixture for subsequent biological experiments. Thus, cells treated with FFAs were categorized as follows: FFAs 0 µm (no FFAs), FFAs 250/125 µm (a mixture containing sodium oleate at a concentration of 250 µm and sodium palmitate at 125 µm), and FFAs 500/250 µm (a mixture of sodium oleate and sodium palmitate at concentrations of 500 and 250 µm, respectively).

### Transmission Electron Microscopy

Cells in the logarithmic growth phase were fixed with precooled glutaraldehyde solution at 4 °C on ice for 2 h, transferred into 2 mL centrifuge tubes, and centrifuged at 10 000 rpm at 4 °C for 5 min. The cell pellets were transferred to vials containing penicillin and washed three times with 4 °C PBS for 15 min each. The cells were then fixed with 1% osmium tetroxide at 4 °C for 30 min and washed three times with PBS. The cells were then dehydrated once with 50% acetone for 10 min, once with 70% acetone for 10 min, twice with 90% acetone for 10 min each, and three times with 100% acetone for 10 min each. Two drops of embedding agent were placed at the bottom of a vial filled with a mixture of embedding agents, and the cell pellet was placed at the center of the vial and baked in a 60 °C oven for 2 h. The embedded surface was removed under a microscope and marked. Using an ultramicrotome, semithin sections (≈1 µm thick) were cut, stained with HE, and observed under a microscope. The paraffin‐embedded slides were placed in a clean culture dish, several drops of sodium acetate stain were added to cover the slides, and the slides were stained for 20–30 min. After staining, the slides were washed three times with double‐distilled water, stained with lead citrate, washed as described, and then observed under a transmission electron microscope (Hitachi H‐7500, Japan) after air drying.

### Indirect Immunofluorescence

TNBC cells (5 × 10⁴ per well) in logarithmic growth phase were seeded onto sterile coverslips in 24‐well plates and cultured overnight (37 °C, 5% CO_2_). Following three PBS washes (3 min each), cells were fixed with 4% paraformaldehyde (15 min) and permeabilized with immunostaining solution (20 min, RT). After blocking with normal goat serum (30 min, room temperature), samples were incubated with primary antibodies (p62 1:150; LC3 1:100) overnight at 4 °C. After PBS washes, Cy3‐conjugated secondary antibody (1:1000) was applied (one hour, room temperature, dark). Nuclei were counterstained with 4',6‐diamidino‐2‐phenylindole (DAPI) (5 min, dark), followed by extensive PBS washing. Coverslips were mounted with anti‐fade medium and imaged using High‐Resolution Confocal Multiphoton Microscopy System (NIKON AX RMP; Japan).

### MTS Assay

Logarithmic‐phase TNBC cells (2.0 × 10⁵ cells/mL) were seeded in 96‐well plates (200 µL per well) and treated with GLPG0974 (5 to 30 nm) for 24 h. After adding MTS reagent (20 µL per well) for 3 h, absorbance was measured at 490/630 nm. Inhibition rate was calculated as [(OD control‐OD treated)/OD control] × 100. IC_50_ was determined via nonlinear regression (GraphPad Prism 10.2.3) from dose‐response curves of (1‐inhibition rate) versus concentration, with triplicate wells and three independent repeats.

### Computational Identification and Validation of High‐Affinity ACAA2 Inhibitors

Using molecular docking and dynamics simulations, we identified potential ACAA2 inhibitors from the Selleckchem L1100‐Inhibitor‐Library (https://www.selleck.cn/screening/inhibitor‐library.html). The ACAA2 structure was predicted via AlphaFold 3 and refined through 100‐ns molecular dynamics simulations (GROMACS 2020.6) to model lactylation effects. Compounds were energy‐minimized and docked against ACAA2 using MOE 2022.02, with the top candidates selected based on docking scores (−10 to −7 kcal mol^−1^), molecular weight (300–500 Da), and solubility. Six lead compounds with binding energies of −10 to −7 kcal mol^−1^ were obtained. Molecular dynamics simulations confirmed stability under physiological conditions (303.15 K, 1 bar) with TIP3P solvation and 0.15 m NaCl. MM‐PBSA binding free energies calculated via gmx_MMPBSA (v1.6.1) further validated the interactions.

### Therapeutic Efficacy Evaluation in Orthotopic Breast Cancer Model

Fifteen tumor‐bearing NKG mice with orthotopically implanted MDA‐MB‐231/ACAA2‐WT cells were randomized into three treatment groups (n = 5 per group): (1) Lotamilast (20 mg kg^−1^, twice weekly via intraperitoneal injection), (2) Fumagillin (30 mg kg^−1^, twice weekly via intraperitoneal injection), and (3) vehicle control. Tumor growth was monitored throughout the treatment period.

### Statistical Analysis

Quantitative data are reported as the mean ± SD (standard deviation) as determined using SPSS 20.0. First, ANOVA was performed, followed by *t‐*tests for pairwise comparisons and tests for homogeneity of variance. In cases of unequal variance, variable transformations or the Kruskal–Wallis test, or the Mann – Whitney test were used. The Chi‐square test, Pearson's correlation coefficient, or Spearman correlation coefficient was used as appropriate to evaluate the correlations between LDHC4 expression and clinicopathological characteristics. Survival curves were constructed with log‐rank tests. The influence of LDHC4 expression levels on the overall survival (OS) of TNBC patients (n = 99) was examined using the univariate and multivariate Cox proportional hazards model. A *p* value of < 0.05 was considered to indicate statistical significance. Graphical representations of the statistical results were created using GraphPad Prism (version 10.2.3).

## Conflict of Interest

The authors declare no conflict of interest.

## Author Contributions

C.Z.L., Z.C.Q., L.Y.Y., and X.Z.Z. contributed equally to this work. C.Z.L., Z.C.Q., L.Y.Y., and P.W. performed the experiments; L.Y.H., W.X.F., and H.S.J. collected clinical samples; C.Z.L., Z.C.Q., and L.A.Y. performed statistical analyses; X.Z.Z. and C.Y. validated results of the experiments; C.Z.L. drafted manuscript and prepared all Figures; S.Y. reviewed the draft manuscript; C.Z.L. and S.Y. contributed to the conception, designed the research, supervised, and edited the manuscript. All authors reviewed the manuscript.

## Supporting information



Supporting Information

Supporting Information

Supporting Information

## Data Availability

The data that support the findings of this study are available from the corresponding author upon reasonable request.

## References

[1] F. Derakhshan , J. S. Reis‐Filho , Ann. Rev. Pathol. 2022, 17, 181. 35073169 10.1146/annurev-pathol-042420-093238 PMC9231507

[2] a) L. Carey , E. Winer , G. Viale , D. Cameron , L. Gianni , Nat. Rev. Clin. Oncol. 2010, 7, 683; 20877296 10.1038/nrclinonc.2010.154

[3] a) A. M. Mahmoud , Immunotherapy 2018, 10, 769; 29926750 10.2217/imt-2017-0179 PMC6462849

[4] a) G. S. Gupta , Mol. Cell. Biochem. 2012, 371, 115; 22893065 10.1007/s11010-012-1428-2

[5] R. Thomas , H. Shaath , A. Naik , S. M. Toor , E. Elkord , J. Decock , Cancer Immunol., Immunother. 2020, 69, 449. 31932876 10.1007/s00262-020-02480-4 PMC7044258

[6] L. Chen , Q. Wu , X. Xu , C. Yang , J. You , F. Chen , Y. Zeng , Exp. Cell Res. 2021, 398, 112414. 33301764 10.1016/j.yexcr.2020.112414

[7] Y. Hua , C. Liang , J. Zhu , C. Miao , Y. Yu , A. Xu , J. Zhang , P. Li , S. Li , M. Bao , J. Yang , C. Qin , Z. Wang , Tumor Biol. 2017, 39, 101042831769596. 10.1177/1010428317695968 28351304

[8] Z. Cui , Y. Li , Y. Gao , L. Kong , Y. Lin , Y. Chen , Aging 2020, 12, 19455. 33035196 10.18632/aging.103879 PMC7732326

[9] W. Peng , J. Chen , Y. Xiao , G. Su , Y. Chen , Z. Cui , Front. Oncol. 2022, 12, 912624. 35814471 10.3389/fonc.2022.912624 PMC9263124

[10] Z. Cui , Y. Chen , M. Hu , Y. Lin , S. Zhang , L. Kong , Y. Chen , Clin. Chim. Acta 2020, 503, 203. 31794764 10.1016/j.cca.2019.11.032

[11] L. Xia , L. Oyang , J. Lin , S. Tan , Y. Han , N. Wu , P. Yi , L. Tang , Q. Pan , S. Rao , J. Liang , Y. Tang , M. Su , X. Luo , Y. Yang , Y. Shi , H. Wang , Y. Zhou , Q. Liao , Mol. Cancer 2021, 20, 28. 33546704 10.1186/s12943-021-01316-8 PMC7863491

[12] a) B. Faubert , A. Solmonson , R. J. DeBerardinis , Science (New York, N.Y.) 2020, 368, aaw5473; 10.1126/science.aaw5473 PMC7227780 32273439

[13] I. Martínez‐Reyes , N. S. Chandel , Nat. Rev. Cancer 2021, 21, 669. 34272515 10.1038/s41568-021-00378-6

[14] a) S. Biswas , C. M. Rao , Eur. J. Pharmacol. 2018, 837, 8; 30125562 10.1016/j.ejphar.2018.08.021

[15] a) P. Ding , Z. Ma , D. Liu , M. Pan , H. Li , Y. Feng , Y. Zhang , C. Shao , M. Jiang , D. Lu , J. Han , J. Wang , X. Yan , Front. Immunol. 2022, 13, 865975; 35585975 10.3389/fimmu.2022.865975 PMC9108232

[16] D. Zhang , Z. Tang , H. Huang , G. Zhou , C. Cui , Y. Weng , W. Liu , S. Kim , S. Lee , M. Perez‐Neut , J. Ding , D. Czyz , R. Hu , Z. Ye , M. He , Y. G. Zheng , H. A. Shuman , L. Dai , B. Ren , R. G. Roeder , L. Becker , Y. Zhao , Nature 2019, 574, 575. 31645732 10.1038/s41586-019-1678-1 PMC6818755

[17] J. Yu , P. Chai , M. Xie , S. Ge , J. Ruan , X. Fan , R. Jia , Genome Biol. 2021, 22, 85. 33726814 10.1186/s13059-021-02308-z PMC7962360

[18] Y. Luo , Z. Yang , Y. Yu , P. Zhang , Int. J. Biol. Macromol. 2022, 222, 2225. 36209908 10.1016/j.ijbiomac.2022.10.014

[19] a) B. Xie , M. Zhang , J. Li , J. Cui , P. Zhang , F. Liu , Y. Wu , W. Deng , J. Ma , X. Li , B. Pan , B. Zhang , H. Zhang , A. Luo , Y. Xu , M. Li , Y. Pu , Proc. Natl. Acad. Sci. USA 2024, 121, 2314128121; 10.1073/pnas.2314128121 PMC10895275 38359291

[20] a) L. Sun , Y. Zhang , B. Yang , S. Sun , P. Zhang , Z. Luo , T. Feng , Z. Cui , T. Zhu , Y. Li , Z. Qiu , G. Fan , C. Huang , Nat. Commun. 2023, 14, 6523; 37863889 10.1038/s41467-023-42025-8 PMC10589265

[21] a) X. Hou , J. Ouyang , L. Tang , P. Wu , X. Deng , Q. Yan , L. Shi , S. Fan , C. Fan , C. Guo , Q. Liao , Y. Li , W. Xiong , G. Li , Z. Zeng , F. Wang , PLoS Biol. 2024, 22, 3002666; 10.1371/journal.pbio.3002666 PMC11192366 38905316

[22] F. Odet , C. Duan , W. D. Willis , E. H. Goulding , A. Kung , E. M. Eddy , E. Goldberg , Biol. Reprod. 2008, 79, 26. 18367675 10.1095/biolreprod.108.068353 PMC2574787

[23] Z. Cui , Y. Li , Y. Lin , C. Zheng , L. Luo , D. Hu , Y. Chen , Z. Xiao , Y. Sun , Front. Endocrinol. 2024, 15, 1328679. 10.3389/fendo.2024.1328679 PMC11109423 38779451

[24] A. Atoui , M. Bou Zerdan , A. El Mahmoud , N. Chamseddine , L. Hamad , H. I. Assi , Int. J. Breast Cancer 2022, 2022, 1218128. 35190777 10.1155/2022/1218128 PMC8858059

[25] D. Miltiadou , A. L. Hager‐Theodorides , S. Symeou , C. Constantinou , A. Psifidi , G. Banos , O. Tzamaloukas , J. Dairy Sci. 2017, 100, 6285. 28624287 10.3168/jds.2016-12326

[26] R. Kanwar , M. Gradzielski , S. Prevost , G. Kaur , D. Clemens , M. S. Appavou , S. K. Mehta , J. Colloid Interface Sci. 2019, 534, 95. 30216837 10.1016/j.jcis.2018.08.066

[27] a) D. V. Pham , P. H. Park , J. Exp. Clin. Cancer Res. 2022, 41, 9 ; 34986886 10.1186/s13046-021-02223-y PMC8729140

[28] Z. Xu , X. Han , D. Ou , T. Liu , Z. Li , G. Jiang , J. Liu , J. Zhang , Appl. Microbiol. Biotechnol. 2020, 104, 575. 31832711 10.1007/s00253-019-10257-8

[29] A. Naik , J. Decock , Mol. Oncol. 2022, 16, 885. 34050611 10.1002/1878-0261.13024 PMC8847988

[30] J. Xiong , J. He , J. Zhu , J. Pan , W. Liao , H. Ye , H. Wang , Y. Song , Y. Du , B. Cui , M. Xue , W. Zheng , X. Kong , K. Jiang , K. Ding , L. Lai , Q. Wang , Mol. Cell 2022, 82, 1660. 35320754 10.1016/j.molcel.2022.02.033

[31] a) K. Yang , X. Wang , C. Song , Z. He , R. Wang , Y. Xu , G. Jiang , Y. Wan , J. Mei , W. Mao , Theranostics 2023, 13, 1774; 37064872 10.7150/thno.82920 PMC10091885

[32] Y. Leng , T. Tian , B. Tang , Y. Ma , Z. Li , Q. Shi , J. Liu , Y. Zhou , W. Wang , C. Huang , X. Zhao , W. Feng , Y. Liu , J. Liang , T. Liu , S. Liu , Q. Ren , J. Liu , T. Zhang , J. Zhou , Q. Huang , Y. Zhang , B. Yin , Y. Xu , L. Liu , L. Shen , H. Zhao , Mol. Carcinog. 2024, 63, 1362. 38656551 10.1002/mc.23729

[33] D. Wu , G. Liao , Y. Yao , L. Huang , B. Dong , Y. Ma , G. Yang , J. Hepatocell. Carcinoma 2023, 10, 1327. 37581093 10.2147/JHC.S418429 PMC10423610

[34] Z. Yang , C. Yan , J. Ma , P. Peng , X. Ren , S. Cai , X. Shen , Y. Wu , S. Zhang , X. Wang , S. Qiu , J. Zhou , J. Fan , H. Huang , Q. Gao , Nat. Metab. 2023, 5, 61. 36593272 10.1038/s42255-022-00710-w

[35] Y. Duan , H. Zhan , Q. Wang , B. Li , H. Gao , D. Liu , Q. Xu , X. Gao , Z. Liu , P. Gao , G. Wei , Y. Wang , Adv. Sci. (Weinh) 2024, 11, 2400227. 39018247 10.1002/advs.202400227 PMC11425215

[36] a) K. Yoshikawa , M. Ishida , H. Yanai , K. Tsuta , M. Sekimoto , T. Sugie , Mol. Clin. Oncol. 2022, 16, 80; 35251631 10.3892/mco.2022.2513 PMC8892429

[37] N. D. Oakes , S. M. Furler , Ann. N Y Acad. Sci. 2002, 967, 158. 12079845 10.1111/j.1749-6632.2002.tb04273.x

[38] a) L. Zhang , L. Han , J. He , J. Lv , R. Pan , T. Lv , J. Cancer Res. Clin. Oncol. 2020, 146, 705; 31773260 10.1007/s00432-019-03095-8 PMC7039835

[39] Y. Zhang , J. Zhao , C. Ren , B. Hu , R. Ding , Z. He , C. Liang , Int. J. Mol. Med. 2023, 51. 10.3892/ijmm.2023.5237 PMC10049036 36928181

[40] A. M. Hossini , X. Hou , T. Exner , B. Fauler , J. Eberle , A. Rabien , E. Makrantonaki , C. C. Zouboulis , Skin Pharmacol. Physiol. 2023, 36, 1. 36384913 10.1159/000527471

[41] a) D. J. Klionsky , G. Petroni , R. K. Amaravadi , E. H. Baehrecke , A. Ballabio , P. Boya , J. M. Bravo‐San Pedro , K. Cadwell , F. Cecconi , A. M. K. Choi , M. E. Choi , C. T. Chu , P. Codogno , M. I. Colombo , A. M. Cuervo , V. Deretic , I. Dikic , Z. Elazar , E. L. Eskelinen , G. M. Fimia , D. A. Gewirtz , D. R. Green , M. Hansen , M. Jäättelä , T. Johansen , G. Juhász , V. Karantza , C. Kraft , G. Kroemer , N. T. Ktistakis , et al., EMBO J. 2021, 40, 108863;

[42] Q. Wu , D. Sharma , Cells 2023, 12, 21287. 10.3390/cells12081156 PMC10136604 37190065

[43] M. Thomas , T. Davis , B. Loos , B. Sishi , B. Huisamen , H. Strijdom , A. M. Engelbrecht , Cell Biochem. Funct. 2018, 36, 65. 29399832 10.1002/cbf.3318

[44] a) J. Chou , D. A. Quigley , T. M. Robinson , F. Y. Feng , A. Ashworth , Cancer Discovery 2020, 10, 351; 32071145 10.1158/2159-8290.CD-19-0528

[45] D. Martínez‐Alonso , M. Malumbres , Semin. Cell Dev. Biol. 2020, 107, 28. 32334991 10.1016/j.semcdb.2020.03.009

[46] A. Loukil , C. T. Cheung , N. Bendris , B. Lemmers , M. Peter , J. M. Blanchard , World J. Biol. Chem. 2015, 6, 346. 26629317 10.4331/wjbc.v6.i4.346 PMC4657123

[47] G. Tchakarska , B. Sola , Cell Cycle (Georgetown, Tex.) 2020, 19, 163. 31885322 10.1080/15384101.2019.1706903 PMC6961668

[48] X. Xiong , L. W. Zheng , Y. Ding , Y. F. Chen , Y. W. Cai , L. P. Wang , L. Huang , C. C. Liu , Z. M. Shao , K. D. Yu , Signal Transduction Targeted Ther. 2025, 10, 49. 10.1038/s41392-024-02108-4 PMC11836418 39966355

[49] J. Xu , Y. Liu , Y. Li , H. Wang , S. Stewart , K. Van der Jeught , P. Agarwal , Y. Zhang , S. Liu , G. Zhao , J. Wan , X. Lu , X. He , Nat. Nanotechnol. 2019, 14, 388. 30804480 10.1038/s41565-019-0381-6 PMC6449187

[50] a) Y. Liu , M. Zhou , M. Xu , X. Wang , Y. Zhang , Y. Deng , Z. Zhang , J. Jiang , X. Zhou , C. Li , J. Controlled Release 2024, 374, 639; 10.1016/j.jconrel.2024.08.045 39208931

[51] T. Bi , Q. Zhao , T. Wang , R. Huang , B. Liu , X. Liu , Y. Wang , Q. Sun , Y. Yang , Z. Liu , Adv. Healthcare Mater. 2025, 14, 2403473. 10.1002/adhm.202403473 39530628

[52] Y. Sun , H. Wang , Z. Cui , T. Yu , Y. Song , H. Gao , R. Tang , X. Wang , B. Li , W. Li , Z. Wang , Drug Resistance Updates 2025, 81, 101248. 40287994 10.1016/j.drup.2025.101248

[53] H. Hong , H. Han , L. Wang , W. Cao , M. Hu , J. Li , J. Wang , Y. Yang , X. Xu , G. Li , Z. Zhang , C. Zhang , M. Xu , H. Wang , Q. Wang , Y. Yuan , Cell Death Differ. 2025, 32, 613. 39753865 10.1038/s41418-024-01436-w PMC11982231

[54] W. Zhu , C. Fan , Y. Hou , Y. Zhang , Cancer Lett. 2025, 627, 217835. 40447130 10.1016/j.canlet.2025.217835

[55] D. Hu , Z. Cui , W. Peng , X. Wang , Y. Chen , X. Wu , Arch. Gynecol. Obstet. 2022, 306, 1185. 35249152 10.1007/s00404-022-06433-3

