# Supplementary material for: Lactate Dehydrogenase C4 Accelerates Triple‐Negative Breast Cancer Progression by Promoting Acetyl‐CoA Acyltransferase 2 Lactylation to Increase Free Fatty Acid Accumulation
Source: Adv Sci (Weinh). 2025 Aug 14;12(40):e11849. doi: 10.1002/advs.202511849 (PMC12561261; doi:10.1002/advs.202511849)
Supplement: Supplementary file 1 — Supporting Information [file ADVS-12-e11849-s002.docx]

| **Supplementary Material Table 1.** Clinical characteristics of LDHC4 expression in TNBC using the validation cohort (n = 99) | | | | | |
| --- | --- | --- | --- | --- | --- |
| Clinicopathological features | Total cases | LDHC4 low (Negative ~ ±) | LDHC4 high (+ ~ +++) | χ2 value | *p* value |
| Age |  |  |  | 2.03 | 0.154 |
| ≥50 | 52 | 13 | 39 |  |  |
| <50 | 47 | 18 | 29 |  |  |
| Location |  |  |  | 0.019 | 0.892 |
| Left | 41 | 15 | 26 |  |  |
| Right | 58 | 22 | 36 |  |  |
| Tumour size |  |  |  | 0.42 | 0.517 |
| >2 cm | 80 | 29 | 51 |  |  |
| ≤2 cm | 18 | 8 | 10 |  |  |
| Histologic grading |  |  |  | 0.367 | 0.545 |
| I–II | 26 | 11 | 15 |  |  |
| II–III | 73 | 26 | 47 |  |  |
| Clinical stage |  |  |  | 2.108 | 0.147 |
| I+II | 69 | 29 | 40 |  |  |
| III+IV | 30 | 8 | 22 |  |  |
| Ki-67 expression |  |  |  | 4.172 | 0.041 |
| ≥14% | 82 | 51 | 31 |  |  |
| <14% | 17 | 6 | 11 |  |  |
| Cancer subtype |  |  |  | 0.006 | 0.936 |
| IDC | 53 | 20 | 33 |  |  |
| NIDC | 46 | 17 | 29 |  |  |
|  | IDC: invasive ductal carcinoma; NIDC: nonspecific invasive carcinoma; LDHC4: lactate dehydrogenase C4 | | | |  |

| **Supplementary Material Table 2.** Clinical characteristics of LDHC4 expression in TNBC (tissue chip, n = 150) | | | | | |
| --- | --- | --- | --- | --- | --- |
| Clinicopathological features | Total cases | LDHC4 low (Negative ~ ±) | LDHC4 high  (+ ~ +++) | χ2 value | *p* value |
| Age | 138*(Data missing, n = 12) |  |  | 3.248 | 0.072 |
| ≥50 | 86 | 45 | 41 |  |  |
| <50 | 52 | 19 | 33 |  |  |
| Location | 150 |  |  | 0.058 | 0.809 |
| Left | 82 | 39 | 43 |  |  |
| Right | 68 | 31 | 37 |  |  |
| Tumour size | 150 |  |  | 0.216 | 0.642 |
| >2 cm | 110 | 50 | 60 |  |  |
| ≤2 cm | 34 | 17 | 17 |  |  |
| Histologic grading | 150 |  |  | 0.2 | 0.654 |
| I–I | 39 | 17 | 22 |  |  |
| II–III | 111 | 53 | 58 |  |  |
| Ki-67 expression | 148*(Missing data n = 2) |  |  | 4.244 | 0.039 |
| ≥14% | 140 | 79 | 61 |  |  |
| <14% | 8 | 1 | 7 |  |  |
| TP53 expression | 132*(Missing data n = 18) |  |  | 0.015 | 0.902 |
| Positive | 88 | 41 | 47 |  |  |
| Negative | 44 | 20 | 24 |  |  |
| EGFR expression | 131*(Missing data n = 19) |  |  | 0.082 | 0.775 |
| Positive | 106 | 50 | 56 |  |  |
| Negative | 25 | 11 | 14 |  |  |
| Cancer subtype | 150 |  |  | 2.726 | 0.256 |
| IDC | 42 | 23 | 19 |  |  |
| NIDC | 96 | 40 | 56 |  |  |
| Others | 12 | 7 | 5 |  |  |
|  | IDC: Invasive ductal carcinoma; NIDC: nonspecific invasive carcinoma; LDHC4: lactate dehydrogenase C4 | | | |  |
